# Metabolic genes on conjugative plasmids are highly prevalent in *Escherichia coli* and can protect against antibiotic treatment

**DOI:** 10.1038/s41396-022-01329-1

**Published:** 2022-10-19

**Authors:** Alana Palomino, Danya Gewurz, Lela DeVine, Ujana Zajmi, Jenifer Moralez, Fatima Abu-Rumman, Robert P. Smith, Allison J. Lopatkin

**Affiliations:** 1 Department of Biology, Barnard College, New York, NY 10027 USA; 2 Department of Biological Sciences, Halmos College of Arts and Science, Nova Southeastern University, Fort Lauderdale, FL 33314 USA; 3 Cell Therapy Institute, Kiran Patel College of Allopathic Medicine, Nova Southeastern University, Fort Lauderdale, FL 33314 USA; 4 Department of Ecology, Evolution, and Environmental Biology, Columbia University, New York, NY 10027 USA; 5 Data Science Institute, Columbia University, New York, NY 10027 USA; 6 Department of Systems Biology, Columbia University, New York, NY 10027 USA; 7 Department of Chemical Engineering, University of Rochester, Rochester, NY 14627 USA

**Keywords:** Antimicrobials, Microbial ecology, Microbiology

## Abstract

Conjugative plasmids often encode antibiotic resistance genes that provide selective advantages to their bacterial hosts during antibiotic treatment. Previous studies have predominantly considered these established genes as the primary benefit of antibiotic-mediated plasmid dissemination. However, many genes involved in cellular metabolic processes may also protect against antibiotic treatment and provide selective advantages. Despite the diversity of such metabolic genes and their potential ecological impact, their plasmid-borne prevalence, co-occurrence with canonical antibiotic resistance genes, and phenotypic effects remain widely understudied. To address this gap, we focused on *Escherichia coli*, which can often act as a pathogen, and is known to spread antibiotic resistance genes via conjugation. We characterized the presence of metabolic genes on 1,775 transferrable plasmids and compared their distribution to that of known antibiotic resistance genes. We found high abundance of genes involved in cellular metabolism and stress response. Several of these genes demonstrated statistically significant associations or disassociations with known antibiotic resistance genes at the strain level, indicating that each gene type may impact the spread of the other across hosts. Indeed, in vitro characterization of 13 statistically relevant metabolic genes confirmed that their phenotypic impact on antibiotic susceptibility was largely consistent with in situ relationships. These results emphasize the ecological importance of metabolic genes on conjugal plasmids, and that selection dynamics of *E. coli* pathogens arises as a complex consequence of both canonical mechanisms and their interactions with metabolic pathways.

## Introduction

Horizontal gene transfer (HGT), specifically plasmid conjugation, plays a major role in microbial ecology and evolution as a dominant way that bacteria can rapidly adapt to their environment. Plasmids are often prevalent in microbial populations and can be transferred between both closely and distantly related phylogenetic groups, providing diverse species access to a common pool of genes [1, 2]. Transferrable plasmids can encode an assorted array of advantageous functions, including protection from antibiotic and xenobiotic compounds, metabolic capabilities such as nutrient utilization, and production of virulence factors, amongst others [3, 4]. Of these, antibiotic resistance genes have gained the most attention [1, 2]. Indeed, plasmid conjugation is considered to be the primary mode for the dissemination of antibiotic resistance to nearly all available antibiotics [5], including aminoglycosides, β-lactams, carbapenems, glycopeptides, tetracyclines, trimethoprims, sulphonamides, and quinolones [6, 7]. These antibiotic resistance genes typically encode for proteins that employ one of three main mechanisms to protect the cell from antibiotic treatment: enzymatic inactivation of the antibiotic (e.g., extended spectrum β-lactamase (ESBL) enzymes or acetyltransferases), reduction in intracellular antibiotic concentrations via altered transport dynamics (e.g., *tetA* efflux pump), or modification/protection of the primary antibiotic target to reduce antibiotic binding (e.g., *sul1* or *qnr* genes) [8, 9].

Antibiotic resistance genes can be associated with common plasmid and/or host-level features, and uncovering these relationships has provided key insights into their dissemination patterns/tendencies. At the strain level, individual antibiotic resistance genes are linked with specific pathogenic clones, molecularly classified by their sequence type (ST). For example, *Escherichia coli* ST131 commonly express ESBL enzymes, and carbapenem resistant *E. coli* (CREC) is often associated with ST167, ST617, ST410, and ST38 lineages [10–12]. Analogously, at the plasmid level, certain incompatibility (Inc) groups have emerged as epidemic amongst *Enterobacteriaceae* [13, 14] due to their association with specific antibiotic resistance genes. For example, aminoglycoside resistance genes (e.g., *aac(6*′*)-Ib-cr*) are more prevalent on IncA/C plasmids [15], whereas the β-lactamase gene *bla*CTX-M-15 has been predominantly found on IncF plasmids [15]. Establishing these trends underlying antibiotic resistance gene prevalence has improved our overall understanding of how resistance spreads through bacterial populations.

In addition to antibiotic resistance genes, conjugative plasmids encode a multitude of diverse functions, including many genes implicated in cellular metabolism. These latter genes are often involved in biosynthetic pathways and/or degradative processes, and can mitigate the energetic demands of stress response [16, 17]. Although these genes are often not the focus of studies focused on conjugative plasmids, several independent works have demonstrated that changing expression levels of genes involved in diverse metabolic and stress processes (henceforth referred to as metabolic genes) can alter antibiotic susceptiblity [18–21]. Moreover, recent work showed that mutations in genes involved in core metabolic processes are clinically prevalent, and over-expressing these genes conferred antibiotic resistance to one or multiple antibiotics, highlighting an underappreciated class of antibiotic resistance mechanisms [20]. Thus, in addition to their primary metabolic function, these findings suggest that metabolic genes on transferrable (e.g., conjugative and/or mobile) plasmids may also protect against antibiotic treatment [19, 21, 22]. However, these secondary implications of metabolic genes are relatively understudied on transferrable elements [23]. Often, plasmids encoding metabolic capabilities are classified as “catabolic”, distinct from resistance plasmids [16, 17]. Overall, the prevalence of these metabolic genes on transferrable plasmids, their relative co-occurrence with canonical antibiotic resistance genes, and their impact on susceptibility phenotypes remain unknown.

To begin addressing this gap, we characterized the prevalence of metabolic genes on transferrable plasmids carried by the representative pathogen *E. coli*, which is notorious for the plasmid-mediated spread of antibiotic resistance. Moreover, we investigated relationships between metabolic gene prevalence and associated plasmid- and strain-level features, along with their co-occurrence (or lack thereof) with canonical antibiotic resistance genes. Finally, our experiments demonstrated that expression of select plasmid-encoded metabolic genes can protect against antibiotic treatment, corresponding to in silico co-occurrence patterns. These results show that the mobile metabolome of *E. coli* may contribute to antibiotic resistance prevalence and highlight the increasing complexity underlying observed antibiotic resistance dissemination patterns.

## Results

### *E. coli* strains and plasmids dataset overview

**Fig. 1:**
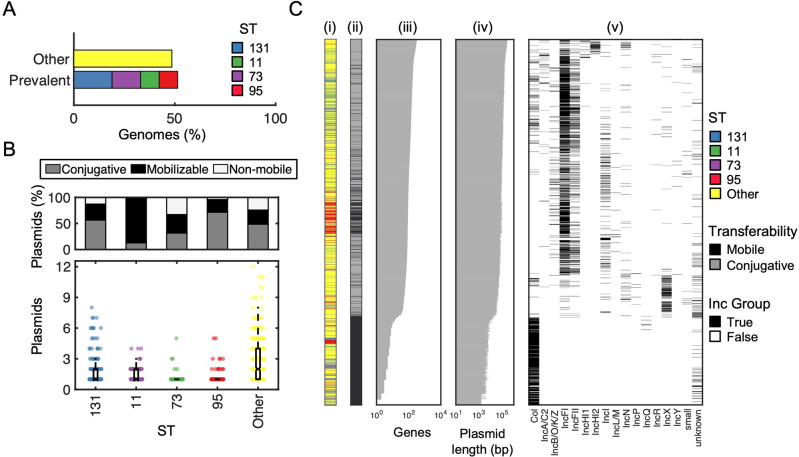
Summary of plasmid dataset and features. **A** ST prevalence in the final dataset. Following a systematic literature search, the dataset consisted of *n* = 823 genomes, wherein 51.4% were made up from the top 4 prevalent STs. **B** Plasmid mobility percentage by ST. *Top*: Percentage of plasmids with either conjugative, mobilizable, or non-mobilizable plasmids. *Bottom:* Number of plasmids per strain categorized by prevalent and non-prevalent ST’s to facilitate interpretation. **C** Plasmid metadata. Multi-panel heatmap consisting of every conjugative and mobilizable plasmid in our final dataset (*n* = 1775, y-axis). From left to right: (i) heatmap indicating ST, where yellow represents non-prevalent and other colors represent prevalent STs; (ii) heatmap indicating plasmid transferability, where black indicates conjugative and gray indicates mobilizable, (iii) bar graph where the x-axis is the number of genes encoded on each plasmid; (iv) bar graph where the x-axis is the plasmid length; (v) heatmap where black indicates the incompatibility group for that plasmid. Plasmids are ordered from greatest to least number of encoded genes (top to bottom).

To investigate the prevalence, range, and correlation of metabolic genes with other strain- and plasmid-level characteristics, we curated a collection of publicly available plasmids associated with a known host (i.e., genome). Specifically, we first retrieved all complete *E. coli* genomes from the NCBI FTP server that contained at least one plasmid sequence (*n* = 823). To ensure this dataset was representative of natural *E. coli* isolates, we also included additional strains based on a systematic literature search (*n* = 1016 total, see Materials and Methods, Table S1). Collectively, a slight majority of this collated dataset (51.4%) was dominated by STs 131, 11, 73, and 95 (Fig. 1A), consistent with trends identified in our literature search, and contained 2235 closed or nearly completed plasmids (see “Materials and Methods”, Table S2). We used MOB-suite to predict plasmid transferability based on the presence of one or more of the following indicators: *ori*T sequence, DNA relaxase, type IV coupling protein, or type IV secretion system [24]. A large majority of plasmids were transferable (i.e., either conjugative (47.1%, *n* = 1052) or mobilizable (32.3%, *n* = 723), Fig. 1B, top, and Fig. 1Cii). Moreover, strains from each ST in our dataset carried fewer than three plasmids on average (Fig. 1B, bottom). This final set of plasmids included 17 incompatibility groups across both clinical and environmental sources, ranged in length from 1538 base pairs to 369,298 base pairs, and contained as few as 1 to as many as 408 genes (*n* = 1775, Fig. 1C, Table S3).

### Plasmid-encoded antibiotic resistance and metabolic genes characterization

We next sought to elucidate the prevalence of both metabolic and canonical antibiotic resistance genes (i.e., those whose primary function is to resist antibiotics by the traditional mechanisms described above, henceforth referred to as antibiotic resistance genes) on these plasmids. Gene annotation revealed 164,697 distinct coding sequences corresponding to 45,962 known genes. 35.1% (*n* = 16,132) of these contained KEGG orthology [20] tags that enabled functional categorization. Specifically, 5.4% (*n* = 2460) of known genes were classified as involved in metabolic processes. Likewise, 9.8% of known genes (*n* = 4483) were classified as encoding antibiotic resistance.

**Fig. 2:**
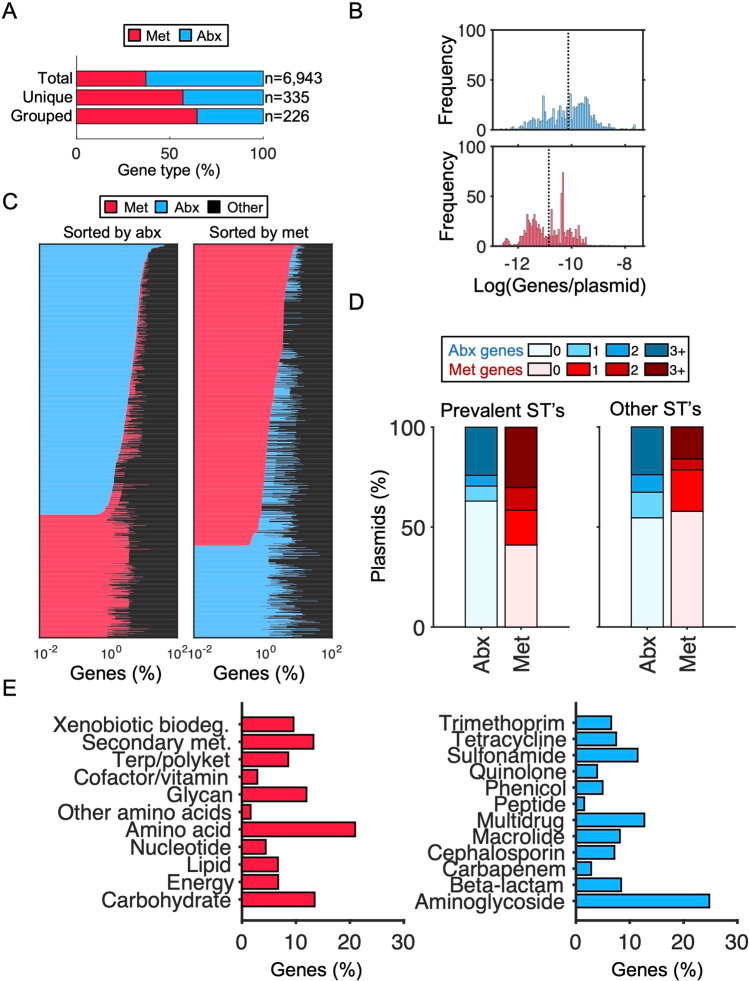
Characterization of antibiotic resistant and metabolic genes. **A** Relative abundance of gene type. Percentage of antibiotic resistance genes (Abx) compared to metabolic genes (Met). Percentages are calculated from the total of the corresponding bar. “Total” corresponds to all annotated antibiotic resistance and metabolic genes, “Unique” corresponds to all unique gene variants, and “Grouped” refers to those from the “Unique” category further grouped by nucleotide variants (e.g., different variants of the same gene are grouped together). **B** Average gene type per plasmid. Histogram distributions show the number of genes (antibiotic resistance or metabolism) per total number of genes on each plasmid. Blue indicates frequency of metabolic genes per plasmid (*top*) and red indicates frequency of antibiotic resistance genes per plasmid (*bottom*). Black dashed line indicates the average number of gene type per plasmid for comparison. **C** Proportion of gene types on plasmids. Left and right panels show stacked horizontal bar graphs where the x-axis is the percentage of genes on each plasmid. Blue indicates antibiotic resistance genes, red indicates metabolic genes, and black indicates all other gene types. Plasmids are sorted top to bottom by antibiotic resistance (*left*) or metabolic (*right*) gene abundance. Any plasmid with neither an antibiotic resistance nor metabolic gene was removed for visibility. **D** Proportion of gene types by ST. Stacked bar graphs show percentage of plasmids with the corresponding number of genes belonging to antibiotic resistance or metabolic gene types. Plasmids are divided into the four categories of encoding 0, 1, 2, or 3+ metabolic genes (shades of red from light to dark) and antibiotic resistance genes (shades of blue from light to dark). Data for prevalent STs (131, 11, 73, 95) and other STs are shown separately (left and right panels, respectively). **E** Percentage of metabolic and antibiotic resistance functions. Red indicates genes belonging to metabolic categories as determined by KEGG (*left*) and blue indicates known antibiotic classes (*right*).

Initially, annotations suggested that antibiotic resistance genes (*n* = 4483) comprised a greater proportion of this collective mobile gene pool, as compared to metabolic genes (*n* = 2460) (Fig. 2A). However, the absolute numbers of unique genes were closer in abundance across classes (191 and 144 unique antibiotic resistance and metabolic genes, respectively). In both cases, these redundancies were primarily driven by repeated occurrences of the same gene on multiple plasmids, rather than multiple copies of the same gene on the same plasmid. Furthermore, grouping gene variants (e.g., *bla*CTX-11 with *bla*CTX-15) reversed this initial relationship altogether: there were more unique grouped metabolic genes (142) than grouped antibiotic resistance genes (84) (Fig. 2A). Finally, although antibiotic resistance genes generally occurred with higher abundances per plasmid (Fig. 2B), metabolic genes occurred on a greater proportion of all plasmids than did antibiotic resistance genes (845 (47.6 %) compared to 752 (42.4%), Fig. 2C). This broader distribution of metabolic genes across plasmids was maintained, and appeared more pronounced, within prevalent sequence types: 59.0% and 37.0% of these plasmids encode metabolic and antibiotic resistance genes, respectively (Fig. 2D). Collectively, these genes encoded a diversity of functions. Among the antibiotic resistance genes, aminoglycoside, multi-class (e.g., efflux pumps), and sulfonamide resistance were most common (Fig. 2E, right). Among the metabolic genes, the most prevalent categories included carbohydrate and amino acid metabolism (Fig. 2E, left).

The percentage of both metabolic and antibiotic resistance genes varied by Inc group (Fig. S2A). In general, and consistent with strain-level trends (Fig. S2B), antibiotic resistance genes comprised notably higher proportions of total gene counts across all Inc groups. We removed “IncQ” from our analysis since these plasmids in total had <5 metabolic genes, and “unknown”, to facilitate interpretation. Of those Inc groups remaining, both metabolic and antibiotic resistance genes were predominately associated with plasmids belonging to IncFI and IncFII (Fig. S2C, *p* < 0.05), potentially suggesting an evolutionary advantage of carrying both on these plasmid types. Although there were more antibiotic resistance genes overall, metabolic genes appeared to be more widely distributed and with higher diversity.

### Significant co-occurrence of antibiotic resistance and metabolic genes

**Fig. 3:**
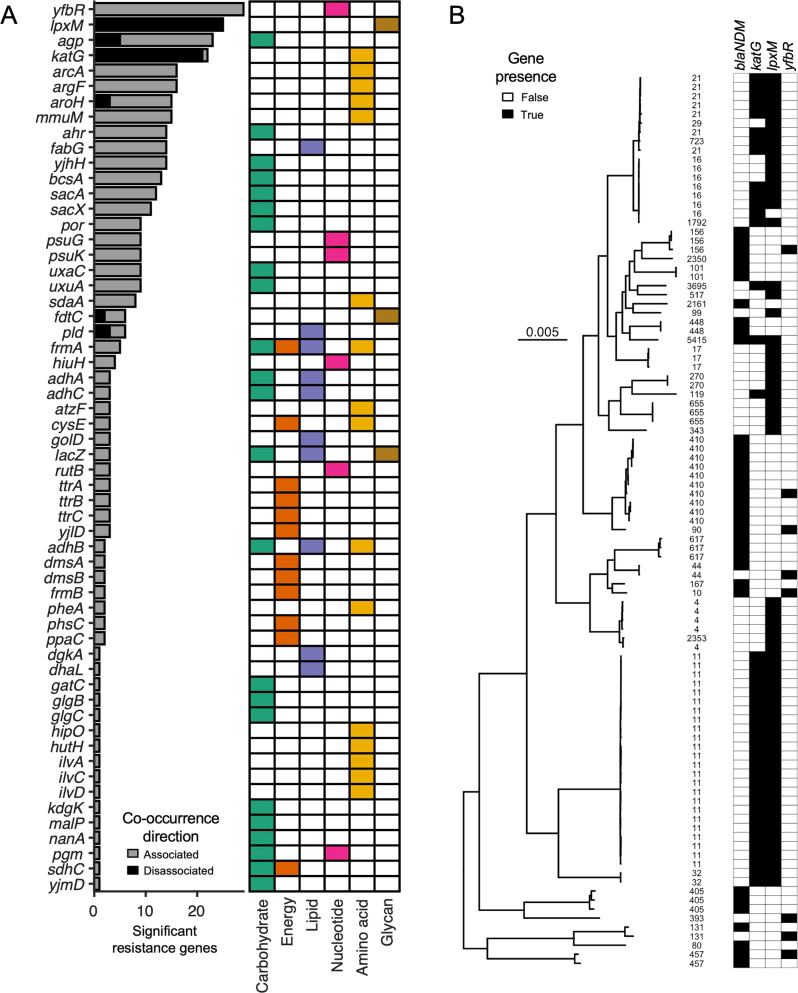
Co-occurrence of metabolic and antibiotic resistance genes. **A** Metabolic gene associations and disassociations with resistance genes. The 57 metabolic genes with statistical relationships to antibiotic resistance genes are ranked in descending order based on the number of associated (gray) and/or disassociated (black) resistance genes (*p* < 0.05). To the right, a heatmap in line with each gene indicates the correspondig KEGG metabolism class: green is carbohydrate metabolism, red is energy metabolism, purple is lipid metabolism, pink is nucleotide metabolism, orange is amino acid metabolism, and brown is glycan biosynthesis. **B** Phylogenetic tree of strains carrying plasmid-encoded *blaNDM*, *katG*, *lpxM* and/or *yfbR*. This tree includes genomes with at least one of the four plasmid-encoded genes, and was filtered for those with a known location, isolation source, isolation date, and clinical origin. The scale bar represents 0.005 nucleotide substitutions per site of the entire aligned core genome. ST corresponding to each tree branch is shown to the left of the gene presence heatmap. Gene presence or absence is indicated by black or white, respectively.

We next looked for statistically significant associations and disassociations between the two gene types, determined by co-location on the same plasmid. To do so, we first ran a random forest out-of-bag predictor model to first understand which general classes across gene types were significantly (dis)associated with each other (Fig. S3A). Based on these results, we removed “peptide resistance” and “other amino acid metabolism” as these did not exhibit significant relationships with any other category. We used the remaining categories to identify antibiotic resistance and metabolic gene pairs whose co-location on the same plasmid was statistically significant. Specifically, 57 metabolic genes were significantly associated, and 6 metabolic genes were significantly disassociated, with at least one antibiotic resistance gene (Fig. 3A, see Table S4A–C). These associations were primarily found on IncF (I and II), IncA/C, and IncN plasmids, but varied depending on the specific pair (Fig. S3B, Table S4D).

### Metabolic genes may restrict the spread of antibiotic resistance genes

Significant disassociations imply specific gene pairs are unlikely to be found on the same plasmid. However, these genes may still co-occur within the same host (i.e., across multiple plasmids), thereby adding complexity to these statistical conclusions. Thus, to better understand gene associations at the strain level, we focused on the three metabolic genes (*katG*, *lpxM*, and *yfbR*) that were among the most commonly implicated with antibiotic resistance genes. Specifically, *yfbR*, which is involved in nucleotide salvage activity [25], exhibited the greatest number of statistical co-occurrences (29 antibiotic resistance genes belonging to nine drug classes) (Fig. 3A). In comparison, *lpxM*, which is involved in glycan biosynthesis [26], was disassociated with 25 antibiotic resistance genes covering nine distinct drug classes. Likewise, *katG*, which counteracts oxidative-stress [27], exhibited disassociative relationships with 21 antibiotic resistance genes belonging to eight drug classes (Fig. 3A, Table S4A–C). Since the oxidative stress response is inextricably linked to bacterial maintenance metabolism [28], we generally categorize *katG* as a metabolic gene for the purposes of this analysis. We used *blaNDM* (all variants) as a representative antibiotic resistance gene, since this gene showed statistical relations with all three of these metabolic genes, and confers resistance to one of the most commonly used antibiotic classes (e.g., β-lactams).

Statistical analysis of gene (dis)associations for *yfbR*, *katG* and *lpxM* at the genome level was consistent with plasmid-level co-occurrences. That is, strains carrying *yfbR* were significantly likely to also carry *blaNDM*, and vice versa for *katG* and *lpxM* (Fisher’s exact test, *p* < 2.5 × 10^−3^ in all cases). These relationships appeared to correlate with evolutionary trends: phylogenetic analysis of representative clinical isolates revealed that genomes containing *katG* and *lpxM* often occurred within clustered lineages and/or distinct clades that were largely separate from those containing *blaNDM* and *yfbR* (Fig. 3B; Fig. S3C). Importantly, gene co-occurrences did not depend on evolutionary history. Statistical (dis)associations were maintained even when correcting for phylogenic effects (*p* < 0.05 in all cases, Table S4E) [29], indicating the presence/absence of these genes across sufficiently diverse clades. Moreover, these trends were maintained at the level of intracellular mobility: most *yfbR* (85%) and *blaNDM* (90%) genes in our dataset were found on or near other plasmid-encoded mobile elements (transposons or insertion sequences), compared to only ~1% of *lpxM* and *katG* (Table S5). Combined, these findings suggest a potential selective advantage or disadvantage for plasmids that simultaneously carry particular metabolic genes and antibiotic resistance accessory genes. For example, over-expression of *katG* was previously found to reduce ampicillin-mediated killing of *E. coli* [30] and has been tied to isoniazid resistance in *Mycobacterium tuberculosis* [31, 32]. That *katG* was significantly independent of numerous antibiotic resistance genes (*n* = 21; Fig. 3A), including *blaNDM*, suggests that *katG* alone may provide a sufficient ecological advantage. Harboring additional resistance mechanisms in this case may be unnecessary and/or prohibitively burdensome.

To investigate the ecological advantages of carrying (dis)associated metabolic genes in the presence or absence of antibiotic resistance genes, we introduced each of *katG, lpxM*, and *yfbR* alone, or co-transcribed with *blaNDM-1*, onto individual medium copy plasmids under the control of an IPTG-inducible promoter. These plasmids, along with *blaNDM-1* alone and an empty plasmid backbone as positive and negative controls, respectively, were transformed into *E. coli*. We refer to the resulting strains as katG, lpxM, yfbR, katG-blaNDM, lpxM-blaNDM, yfbR-blaNDM, blaNDM, and ctrl for simplicity (Table S6). We confirmed IPTG induction by measuring the minimum inhibitory concentrations (MIC) of blaNDM to carbenicillin (Fig. S4). We then carried out pairwise competition experiments between IPTG-induced katG and katG-blaNDM, lpxM and lpxM-blaNDM, and yfbR and yfbR-blaNDM, and quantified relative fitness of the single gene compared to its co-expressed version. Consistent with our phylogenetic analysis, both katG and lpxM outcompeted their -blaNDM co-expressing counterparts, whereas yfbR alone had no fitness advantage, and in some cases was outcompeted by yfbR-blaNDM (Fig. S5A–C). This finding held true in multiple media types and background strains (Fig. S5D, E). Overall, these results illustrate that observed genetic (dis)associations may be driven by the resultant ecological fitness.

### Disassociated metabolic genes protect against antibiotic treatment

**Fig. 4:**
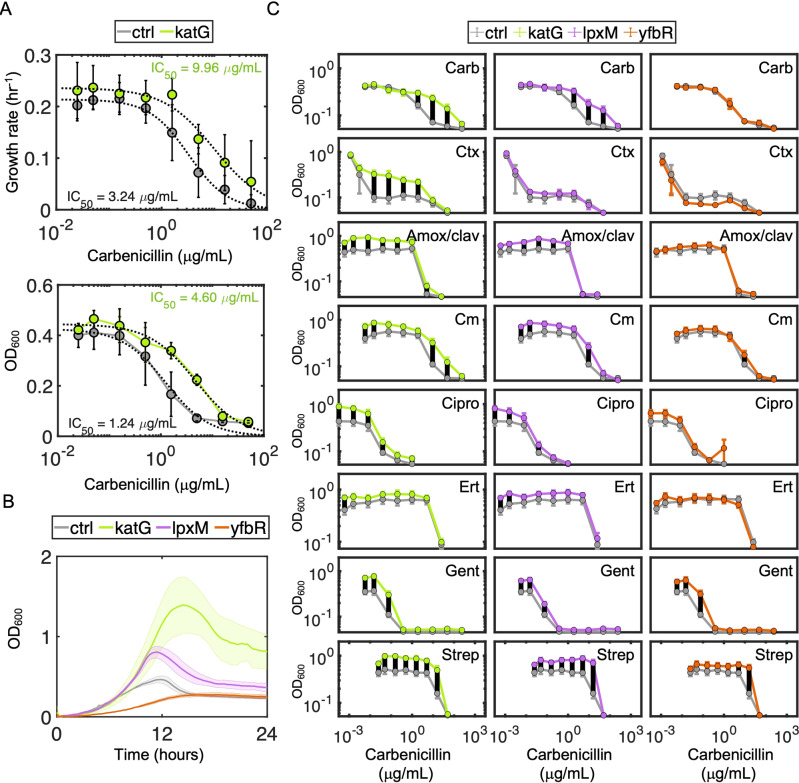
Metabolic genes protect against antibiotic treatment. **A** Carbenicillin dose responses. Dose responses of the ctrl (gray) and katG (green) strains were measured using either growth rates (*top*, y-axis), or optical density after 20 h (*bottom*, y-axis), in the presence of varying concentrations of carbenicillin: 50, 15.81, 5.0, 1.58, 0.50, 0.158, 0.05, and 0 μg/mL (x-axis). IC_50_’s were determined by fitting growth rate estimates or OD_600_ measurements as described in Materials and Methods. Data points represent the mean of at least four biological replicates, and error bars represent the standard deviations. **B** Representative temporal dynamics. Representative timecourses are shown for all three metabolic strains and ctrl treated with 1.58 μg/mL carbenicillin. Shaded error bars represent the 95% confidence interval of three technical replicates. X-axis is time in hours, and y-axis is log-transformed OD_600_. The colors gray, green, purple, and orange, correspond to ctrl, katG, lpxM, and yfbR, respectively. **C** Density-based dose responses. Density after 20 h for ctrl (gray), katG (green), lpxM (purple), and yfbR (orange) was determined for at least six biological replicates per strain for each antibiotic: carbenicillin (Carb), ceftriaxone (Ctx), amoxacillin/clavulanic acid (Amox/clav), chloramphenicol (Cm), ciprofloxacin (Cipro), ertapenem (Ert), gentamicin (Gent), and streptomycin (Strep), respectively. Error bars represent the standard error, across all replicates. The x-axis is antibiotic concentrations in μg/mL and y-axis is OD_600_. Black lines between curves indicate data points where the metabolic gene was statistically greater than the control strain (*p* < 0.05, right-tailed *t* test).

Given the prevalence of metabolic genes on conjugative plasmids, that expression of metabolic genes can impact antibiotic resistance phenotypes [20], and that expression of these genes alone can confer a fitness advantage (e.g., Fig. S5), we wondered to what extent the expression of *katG*, *lpxM*, and *yfbR* might impact antibiotic susceptibility in the absence of canonical antibiotic resistance genes. To determine changes in antibiotic susceptibility, we first measured IC_50_ values, which captures both growth inhibition and overall survival, in response to the representative β-lactam carbenicillin. katG exhibited a significantly higher carbenicillin IC_50_ compared to ctrl (~3.5-fold increase or more), regardless of whether IC_50_ was quantified using the maximum growth rate (Fig. 4A, top), steady state densities at 20 (Fig. 4A, bottom), 14, 16, or 24 hours (Fig. S6A), over the entire time course (Fig. 4B), or with/without mineral oil (Fig. 4C top row, Fig. S6B). Similar to katG, lpxM exhibited improved growth under carbenicillin treatment compared to ctrl, whereas yfbR was more sensitive. To test whether these results were specific to carbenicillin, we next measured susceptibility to ceftriaxone, which belongs to the closely related cephalosporin antibiotic class. Consistent with carbenicillin, katG exhibited increased resistance to ceftriaxone compared to ctrl (Fig. 4C, second row). Although dose responses did not show a clear change in susceptibility for lpxM, both lpxM and katG exhibited small increases in their MIC (Fig. S7A), suggesting that both could confer modest resistance, which we confirmed with colony forming units (CFU) (Fig. S7B). In contrast, yfbR did not confer a susceptibility advantage under ceftriaxone treatment (Fig. 4C). Expanding these results to eight drugs representing the most commonly used antibiotic classes confirmed small shifts in sensitivity. However, for a majority, both katG and lpxM demonstrated higher steady-state densities in subinhibitory concentrations. In contrast, yfbR did not benefit from any selective advantage in the presence of these antibiotics (Fig. 4C).

**Fig. 5:**
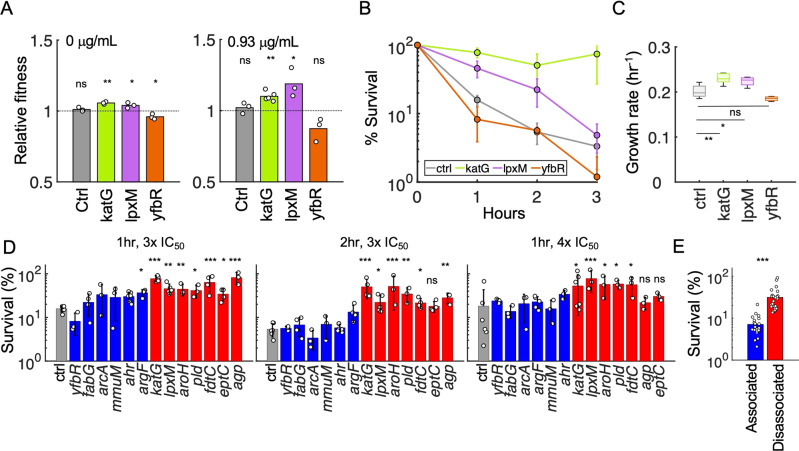
Mechanistic intuition and generality. **A** Relative fitness for (dis)associated genes. Competition experiments between ctrl-cmR (strain 19 denoted S19 in Table S6B) and ctrl-kanR (S2), katG (S4), lpxM (S5), or yfbR (S6) in the absence (*left*) or presence (*right*) of 0.75x IC_50_ (0.93 μg/mL) carbenicillin (see Table S12 for details). Relative fitness was calculated as the fold change in log cell density ratio over 24 h where M is the kanR version of each strain and W is the cmR version of each strain as described in “Methods”. Averages are shown for three independent biological replicates. *p* values are calculated based on the significant difference from neutral fitness (i.e., 1) using one-way t tests. cmR and kanR refer to chloramphenicol and kanamycin resistance, respectively. **B** Time-kill kinetics for representative genes. Ctrl, katG, lpxM, and yfbR survival over time is measured in the presence of 3x IC_50_ (3.75 mg/mL) carbenicillin. Survival percentage is calculated by normalizing the CFU at every time point by the CFU at time = 0 and multiplying by 100. Error bars are standard deviation of at least three individual biological replicates. **C** Growth rates in minimal media of (dis)associated genes. Box plots consist of at least three independent biological replicates. **D** Time-kill kinetics for all 13 genes. Survival over time is measured for ctrl (gray), six associated (blue: *yfbR*, *agp*, *fabG*, *arcA*, *mmuM*, and *ahr*) and six dissociated (red: *katG*, *lpxM*, *aroH*, *pld*, *fdtC*, and *eptC*) genes in the presence of 3x IC_50_ (3.75 mg/mL) carbenicillin after 1 (*left*) and 2 (*middle*) hours of treatment, or 4x IC_50_ (5 mg/mL) carbenicillin after 1 (*right*) hour of treatment. Percent survival is calculated by normalizing the CFU at every time point by the CFU at time = 0 and multiplying by 100. Error bars show standard deviation of at least three individual biological replicates. See Table S14 for statistics. **E** Disassociated genes protect against carbenicillin treatment. Data from the middle panel of (**D**) was pooled together for each unique relationship type. Strains expressing disassociated genes have statistically greater survival than associated genes (two-tailed t test). One, two and three asterisks indicate *p* < 0.05, 0.01, and 0.001, respectively, and “ns” is not significant, where applicable. All *p* values from panels with multiple *t* tests were Bonferroni corrected.

Combined, these results suggest that harboring multiple resistance pathways (i.e., via both gene types) may, in some cases, be evolutionarily disadvantageous: *katG* and *lpxM* (which often exhibited improved fitness under antibiotic treatment) were primarily *disassociated* with antibiotic resistance genes, whereas *yfbR* often conferred negligible advantage and was *associated* with antibiotic resistance genes. To confirm whether these absolute fitness effects translated into relative fitness (dis)advantages overall, we conducted pairwise competition experiments between katG, lpxM, and yfbR, each against ctrl. Indeed, katG and lpxM outcompeted ctrl, in both the absence (Fig. 5A, left, *p* < 0.05) and presence of sub-inhibitory carbenicillin (Fig. 5A, right, *p* < 0.05), confirming their relative fitness advantage. On the other hand, yfbR did not grow significantly better than ctrl in either case (*p* > 0.1). Although *katG* and *lpxM* are functionally unrelated, and therefore likely rely on distinct mechanisms to impart antibiotic protection, this observation suggests some initial intuition: while growth rates and killing rates are often directly correlated, their relationship can be disrupted when metabolism becomes more efficient (i.e., more growth from the same/lower energy expenditure) [33]. Thus, since katG and lpxM exhibited both relative and absolute fitness advantages, even in the absence of drug treatment, this suggests that protection from carbenicillin may arise due to improved growth with negligible changes in metabolic activity, ultimately minimizing antibiotic-mediated killing rates. Indeed, time-kill curves revealed that both katG and lpxM were killed more slowly and survived to a greater extent than ctrl, whereas yfbR survival was indistinguishable from ctrl (Fig. 5B). Moreover, individual growth curves confirmed higher growth rates for katG and lpxM in minimal (Fig. 5C) and rich (Fig. S8A) media, and no significant changes in oxygen consumption rate (OCR) (Fig. S8B). In contrast, yfbR exhibited no change in growth rate (Figs. 5C and S8A) accompanied by a reduction in OCR (Fig. S8B). Together, this is consistent with previous findings highlighting that increased metabolic efficiency can protect cells from antibiotic treatment [33].

Our results suggested that strains expressing disassociated genes exhibited higher growth and reduced killing in the presence of antibiotics. In contrast, growth and antibiotic susceptibility of strains expressing associated genes was no different than the control strain. To examine the generality of these findings, we investigated an additional five associated (*fabG*, *arcA*, *mmuM*, *argF*, and *ahr*) and five disassociated (*agp*, *aroH*, *pld*, *fdtC*, and *eptC*) genes from our pairwise gene analysis (Fig. 3A; see Table S6C for gene descriptions). Although *eptC* was not one of the six initial disassociated genes identified, a secondary analysis examining metabolic gene associations with antibiotic classes rather than specific resistance genes, revealed significant disassociations between this gene and β-lactam antibiotics (*p* = 2.3 × 10^−13^), and thus was included for completeness. Consistent with our expectations, all seven disassociated genes exhibited higher growth rates (Fig. S8D), and reduced killing (Fig. 5D, E, Table S13), than ctrl, whereas associated genes’ growth rates (Fig. S8C, Table S13) and killing remained largely unchanged compared to ctrl (Fig. 5D, E). Collectively, this paradox of faster growth and improved survival runs counter to established dogma that, all else being equal, faster growing cells are preferentially killed by antibiotics due to their elevated metabolic activity. Indeed, *katG*, *IpxM*, and other metabolic genes disassociated from antibiotic resistance genes may confer observed advantages via metabolic dysregulation that enables faster growth while escaping drug-mediated killing.

## Discussion

Studies have primarily focused on characterizing genes encoding canonical resistance mechanisms and their ability to spread on plasmids via HGT. Our knowledge of analogous metabolism-related genes involved in antibiotic resistance, and their extent, diversity, and spread has been limited. Here, we leveraged an extensive collection of publicly available genomes to characterize metabolic genes found on transferrable *E. coli* plasmids. We found that metabolic genes occurred, in most cases, with comparable frequency compared to antibiotic resistance genes, but their relative prevalence varied by genomic context (e.g., ST, etc.). Analogous to categorizations established for antibiotic resistance genes, certain metabolic functions were significantly correlated with common incompatibility groups (e.g., IncF). Moreover, their plasmid-level associations varied by ST. Our analysis was limited to plasmid-level associations. These findings can be readily expanded to examine genomic-level associations, although careful consideration should be taken to define core and accessory gene groups given likely redundancies in metabolic functions and pathways.

Specific metabolic genes exhibited significant associative and/or dissociative relationships with known antibiotic resistance genes. Experiments illustrated that carrying certain metabolic genes may confer selective advantages in the presence of one, and in some cases several, antibiotics. In other words, antibiotic resistance phenotypes may arise as potential secondary effects of acquired metabolic functionality. Specifically, expressing *yfbR* tended to confer little to no resistance compared to the control strain, whereas expressing *katG* and *lpxM* demonstrated a notable survival advantage at lower antibiotic concentrations for many antibiotic classes, and higher concentrations of carbenicillin and ceftriaxone. These phenotypic results are consistent with our bioinformatic analysis, wherein *katG* and *lpxM*, but not *yfbR*, were often found in the absence of antibiotic resistance genes.

We note that environmental factors likely play a role in altered susceptibility conferred by metabolic variants. For example, strains carrying *lpxM* exhibited a 2-fold higher ceftriaxone MIC in LB media, compared to the control strain. However, no shifted dose response was apparent in minimal medium. Indeed, the ecological and evolutionary fates of conjugative plasmids are likely dependent on antibiotic conditions, nutrient availability, and resource consumption characteristics, amongst other factors. Environmental conditions were held constant throughout the present study. Exploring the impact of these factors represent a key future layer of complexity.

Although we observed conserved growth/metabolic trends, we expect unique mechanisms to explain the susceptibility phenotypes for each disassociated gene. For example, highly expressed *katG* likely reduces antibiotic lethality by mitigating toxic metabolic dysregulation following bactericidal drug treatment. In contrast, the role of *lpxM* in outer wall and membrane metabolism suggests more drug-specific and/or transport pathways. Moreover, mechanisms underlying these co-occurrences, or lack thereof, are likely more complex than intracellular relationships alone. For example, functions that provide communal benefits (e.g., β-lactamase genes) may extend to inter-species interactions in mixed populations. However, in all cases, future research is necessary to elucidate the molecular details of each, as well as why certain genes work better in conjunction with antibiotic resistance genes, than others.

Regardless of the gene-specific mechanisms, our results establish an initial ecological explanation for the observed evolutionary (dis)associations: that metabolic-antibiotic resistance gene combinations can be either advantageous or detrimental highlights the co-occurrence of these gene types have nuanced effects on overall fitness. Indeed, classic antibiotic resistance genes undoubtedly confer significantly greater protection to lethal drug treatment than metabolic genes (e.g., *blaNDM-1* compared to *katG*). However, that the combination of these two gene types may be sufficiently detrimental to constrain both genes’ evolutionary dissemination patterns (e.g., Fig. 3B) suggests that even plasmids without canonical resistance genes may play a greater role than previously thought in the spread of antibiotic resistance. Such insights have long-term implications in both better predicting antibiotic resistance evolution, as well as alternative antimicrobial approaches that leverage ecological dynamics [34].

Overall, our results suggest that observed plasmid-mediated antibiotic resistance may arise as a function of metabolic variants as well as canonical resistance mechanisms. These results suggest that antibiotic treatment may lead to simultaneous and/or synergistic selection for both canonical and metabolic resistance mechanisms. A broader functional characterization of these metabolic genes, both in isolation and in conjunction with antibiotic resistance pathways, is critical to better understand how metabolic adaptation can lead to, potentiate, or in some cases, inhibit antibiotic resistance dissemination. Our findings focused on *E. coli*. However, plasmid-mediated resistance spread is important in other bacterial species, including clinically relevant *Enterobacteriaceae* such as *Klebsiella pneumoniae*, and potential environmental contributors such as *Pseudomonas spp*. In addition to their clinical relevance, these future investigations could demonstrate the broad applicability of metabolic genes found in this study and suggest potential novel antibiotic targets to prevent antibiotic resistance dissemination.

## Materials and methods

### Retrieval of E. coli plasmid sequences

All *E. coli* sequences were downloaded from the NCBI FTP server in May 2020. To establish an initial collection of plasmids, only complete genomes with an associated plasmid were retained. All genomes were verified for belonging to the species *E. coli* using kmerfinder (https://cge.cbs.dtu.dk/services/KmerFinder/). Sequence type (ST) was determined via multi-locus sequence typing (MLST) based on the 7-gene Achtman scheme using pubMLST (https:/github.com/tseemann/mlst). Only genomes with exact matches were assigned for each ST and used for subsequent analysis. To ensure our sequences were sufficiently representative of *E. coli* pathogens expected in nature, a systematic literature search (see description below and Fig. S1) was conducted to establish an expected distribution of STs (Table S1). This information was used to update our initial collection to match the top 4 most prevalent STs (131, 11, 73, and 95). Specifically, to identify supplementary plasmid sequences, genome accession IDs were chosen from EnteroBase based on the following criteria: the strain was matched to the correct ST and had a high-quality genome sequence (based on N50 > 20,000 and the number of contigs <250). Of those that met our criteria, genomes were randomly chosen until our desired distributions were achieved. These genome sequences were downloaded from GenBank and plasmids were assembled using plasmidSPAdes [35] (*n* = 1044). Assembled plasmids with an identifiable incompatibility group and at least 95% percent identity to any plasmid on NCBI via BLAST were then added to our initial plasmid collection. Finally, genomes were filtered to remove those without a known high quality phylotype (*n* = 1016), establishing a plasmid-level dataset consisting of 2235 (1984 complete and 251 assembled) unique plasmids, and 1775 unique transferrable plasmids (excluding non-mobilizable plasmids). The complete list of all plasmids can be found in Table S2.

### Systematic literature search

A comprehensive literature review was conducted to establish a representative distribution of *E. coli* ST’s expected in nature (Fig. S1). In particular, a search using the databases PubMed and EMBASE was performed to retrieve primary literature using the following key search terms: “Escherichia coli”, “sequence type”, “population structure”, and “antibiotic resistance”. Search terms were limited to publications in the English language from January 1st, 2008, through present (as of July 2020). The inclusion criteria for articles were as follows: sequence type frequencies were described, and total number of isolates reported. Articles were also required to include at least 10 isolates. Articles were excluded if there was no clear information on isolate source or collection. Total sample number and frequency of each sequence type mentioned were recorded (Table S1). Data was also collected on isolate source (e.g., human patients, animal, or environmental sources).

### Plasmid annotation

Plasmids were annotated using Prokka [36], which provided KEGG [37] gene identification tags, along with GenBank, and manually curated to verify consensus and mismatching genes. To verify the presence of antibiotic resistance genes, plasmids were additionally annotated using the CARD database (https://card.mcmaster.ca/), AMRfinder [38] and ResFinder [39] tools. A consensus from at least two sources, or the results from ResFinder otherwise, was used to update the existing annotations. Incompatibility groups were determined using PlasmidFinder [40], and plasmid mobility was determined based on established mobility typing schemes using MOB-typer [24]. Genes from each plasmid were then combined with plasmid-level metadata using a custom MATLAB pipeline that integrated KEGG classification categories and resistance gene counts per plasmid. The resulting merged dataset contains gene-level information per unique plasmid as well as important plasmid-specific metadata (Table S3). Gene associations with mobile elements were determined using MobileElementFinder. All gene types were determined to be associated with a mobile genetic element if they were found within 30 kb of the mobile element sequence [41]. Results were compiled based on identifying mobile elements with 100% percent identity and coverage (Table S5).

### Phylogenetic analysis

To generate the clinical genome phylogeny, all non-clinical genomes were removed, along with any strain that did not carry a transferrable plasmid (Fig. S3C). To generate a representative phylogeny, the same subset of genomes was further filtered to remove those with unknown collection dates or locations (Fig. 3B). In all cases, core genomes were aligned using Roary [42], and phylogenetic relationships were inferred using RAxML [43]. Trees were visualized and annotated in R using the ggtree [44–46] library.

### Creation of over-expressed metabolic and antibiotic resistance genes

Genomic DNA was extracted from *E. coli* strain MG1655 using a ZR Fungal/Bacteria DNA Miniprep kit (Zymo Research, Irvine, CA) kit following manufacturer’s directions. All primers can be found in Table S6A. Genes *katG*, *lpxM*, *aroH*, *arcA*, *mumM, apg*, *agrF*, *ahr*, *fabG*, *eptC* and *yfbR* were amplified from genomic DNA. Genes *pld* and *ftdC* were synthesized as GeneBlocks (IDT DNA, Coralville, IA) and were then amplified. *bla-NDM-1* was amplified from the plasmid pGDP1 NDM-1 (Addgene, Watertown, MA), which was extracted using a Zyppy Plasmid Miniprep kit following manufacturer’s directions. We used Q5 High-Fidelity DNA Polymerase (New England BioLabs, Ipswich, MA) for all PCR reactions using the following cycling protocol: 95 °C for 5 min, followed by 30 cycles of 95 °C (30 s), 61 °C (30 s) and 72 °C (3 min) followed by 5 min at 72 °C. All amplicons were gel purified using NucleoSpin Gel and PCR clean up kit (Takara Bio, Mountain View, CA). When cloning *yfbR*, *lpxM*, *arcA*, *mumM*, *ahr, ftdC*, *pld*, *eptC* and *katG* amplicons, both amplicons and plasmid pPROLAR were cut with Kpn1 and HindIII (New England Biolabs). When cloning *bla-NDM*-1, *aroH*, and *fabG* both the amplicon and pPROLAR were cut with EcoR1 and HindIII (New England Biolabs). When cloning *agp* and *agrF*, both the amplicon and pPROLAR were cut with Kpn1 and Xba1 (New England Biolabs). Cut amplicons were purified using a QIAquick PCR purification kit (Qiagen, Germantown MD) following manufacturer’s recommendations. Ligation occurred overnight at 16 ^o^C using T4 DNA ligase (New England Biolabs). Competent *E. coli* DH5αPRO cells were prepared and transformed using a Mix & Go! *E. coli* Transformation Kit following the manufacturer’s recommendations. Transformants were selected overnight on LB agar containing 50 μg/mL kanamycin. Sequences were verified using Sanger sequencing. To create *blaNDM-1*-gene fusions (*blaNDM-lpxM, blaNDM-katG, blaNDM-yfbR*), we extracted pPROLAR containing *blaNDM-1* from sequence verified DH5αPRO bacteria. *katG*, *lpxM*, and *yfbR* amplicons were produced following the protocol described above using fusion primers listed in Table S6A. Note that the forward primer of each primer pair contained the same ribosomal binding sequence as that driving *bla-NDM-1* to ensure comparable translation rates. Both *blaNDM*-*1* + pPROLAR and the resulting amplicons were cut with HindIII. To reduce recircularization of the plasmid, *blaNDM*-*1* + pPROLAR was treated with Quick CIP (New England Biolabs). Purification of plasmid and cut amplicons were completed using a QIAquick PCR purification kit. Ligation, transformation and selective plating occurred as described above. The orientation of metabolic genes relative to *blaNDM-1* was initially verified using restriction digest, and subsequently confirmed using Sanger sequencing. To create chloramphenicol resistant versions of *blaNDM-lpxM*, and *blaNDM-yfbR*, the kanamycin resistance marker was replaced with the chloramphenicol resistance marker from pPROTET using AatII and Spe1. Relevant bands were gel excised and purified as described above. To create chloramphenicol resistant *blaNDM-katG*, the chloramphenicol resistance marker was first cloned into pPROLAR containing *blaNDM*. *katG* was then cut from the plasmid contained in the katG strain and inserted using HindIII. CIP was used as described above to prevent recircularization of cut plasmids. Transformants were selected overnight on LB agar containing 25 μg/mL chloramphenicol. We confirmed the presence of all genes using PCR.

### General bacterial growth conditions

In all cases, the strain DH5αPro was transformed with the plasmid of interest (Table S6B). Strains were streaked onto Luria-Bertani (LB) (BD Difco, catalog #DF0446-07-5) agar plates containing the appropriate antibiotic (50 μg/mL for kanamycin, 30 μg/mL for chloramphenicol, and 50 μg/mL for rifampicin). For every experiment, individual colonies were picked and inoculated into 2 mL LB broth and grown for exactly 16 h at 37 °C with 250 rpm agitation, along with a LB-only negative control. Cultures were used the following day only if the negative control was clear.

### Measuring the MIC

All strains were grown as previously described. After 16 h, cells were diluted 1:1000 in LB media with 1 mM IPTG and 50 μg/mL kanamycin, and placed at 25 °C for one hour to initiate gene expression. Diluted cells were then aliquoted into wells of a 96 well plate. Antibiotics were added to the leftmost well to achieve the highest concentration of the respective antibiotic (256 and 16 for carbenicillin and ceftriaxone, respectively), and serially diluted two-fold from column 2 until column 11. Sterile water was added to column 12 instead of antibiotic as a growth control, and the total final volume in each well was kept at 100 μL. Plates were then sealed with a paper film (BioExcell, catalog # 41061023), covered with the plastic lid, and incubated in a shaking 37 °C incubator for 20 h at 250 rpm. Following incubation, cell growth was measured by optical density (OD) in an absorbance Tecan MPLEX plate reader at 600 nm. For each condition, at least two technical replicates were averaged to determine one biological replicate value. The MIC was determined by identifying the first concentration of the antibiotic that resulted in an OD_600_ value which did not statistically differ (*p* < 0.05) from the cell-free LB control (~0.05 OD_600_), using a student’s *t* test. All experiments were conducted with at least three independent biological replicates. To confirm IPTG induction, the MIC as described above using blaNDM with, and without, 1 mM IPTG in the growth media (Fig. S4A, Table S8). After establishing that IPTG significantly increased the MIC of blaNDM compared to the IPTG-free control, shifts in MIC were determined by comparing each strain’s MIC to ctrl, in the presence of IPTG (Fig. S4B, Fig. S7, Table S9).

### Quantifying 20-h sensitivity for all antibiotics

Analysis of carbenicillin time course curves with carbenicillin revealed that changes in antibiotic sensitivity according to the IC_50_ values were equivalent depending on whether IC_50_ was quantified using the growth rates of the entire time courses, or the final density at 14, 16, 20, and 24 h (Fig. S6A, Fig. 4A bottom). Thus, to streamline analysis and increase experimental throughput, antibiotic susceptibility for the remaining 7 antibiotics was quantified using 20 h OD_600_ measurements instead of growth rates. In particular, experiments were set up analogously as described above, except plates were sealed with a paper seal and incubated in a shaking 37 °C incubator for 20 h at 250 rpm, instead of covering wells with mineral oil (Fig. S6B, Fig. 4C top row). We note that inter-day variability in susceptibility responses were occasionally observed (Table S10), likely due to environmental fluctuations. Although a minority of replicates did not exhibit shifted carbenicillin sensitivity, sufficient replicates ensured this heterogeneity did not affect the trends described.

### Confirming OD shifts using colony forming units (CFU)

To verify that changes in MIC as determined by OD_600_ were indeed due to differences in cell number rather than morphology, cell debris, or other OD_600_ artifacts, we quantified CFUs under conditions that exhibited a clear shift in MIC in response to ceftriaxone. Specifically, the MIC of katG was determined to be 0.125 μg/mL, while ctrl had no observable growth at this concentration (and instead showed an MIC of 0.0625 μg/mL). To confirm the growth of katG at higher antibiotic concentrations was real, the colony forming units (CFU) of both strains at 0.125 μg/mL ceftriaxone was obtained following a standard MIC experiment. After 20 h of incubation, cells were serially diluted, and spot plated using 10 μL in 3 technical replicates on LB agar containing 50 μg/mL kanamycin. CFUs were averaged for all technical replicates and conducted for two biological replicates for each strain (Fig. S7B, Table S11).

### Quantifying growth rates and IC_50_ values

All strains were grown as previously described. After 16 h, each clone was resuspended and diluted 1:1000 in the appropriate media. In the text, “minimal” media refers to M9 minimal media with casamino acids, 0.4% glucose (M9CA medium broth catalog with 1 mM thiamine (cat #J864-100G). 40% glucose solution (cat #G2020), referred herein as M9CAG), and “rich” refers to standard LB media. In all cases, media was supplemented with 50 μg/mL kanamycin and 1 mM IPTG. Clones were then aliquoted into wells of a 96-well microtiter plate to achieve a total volume of 200 μL per well. In the case of IC_50_ measurements, log serial dilutions were prepared for each antibiotic and aliquoted to achieve the desired final concentrations (Table S7). Wells were covered in 50 μL mineral oil and growth was measured by absorbance in a Tecan MPLEX plate reader at 600 nm in 15-minute intervals for up to 24 h. To analyze the time courses, growth curves were first background subtracted and log-transformed in all cases. The maximum growth rate μ_m_ was calculated using the modified logistic equation [47]:

$$N = \frac{A}{{\left[ {a + e^{\frac{{4\mu _m}}{A }\left( {\lambda - t} \right) + 2}} \right]}}$$

for all experiments measured in minimal media, referred to as “Logistic method”. For experiments in rich media, growth rates were determined by obtaining the maximum derivative within the longest region associated with linear growth. This region of the growth curve was identified using k-means clustering with 3 groups to separate lag, log, and residual growth/stationary phases, as described previously [48]. This separate method, referred to as the “slope method”, was used for growth in rich media since, upon inspection, the multi-phase growth that is characteristic of LB media reduced the quality of logistic fits in many cases. Regardless, both methods resulted in trends that were qualitatively and statistically consistent for rich media data (Fig. S8A left compared to right). Therefore, growth rates for all genes (Fig. S8D) were measured in rich media and quantified using the slope method. To calculate the IC_50_ values, growth rates (μ_m_) were determined at each antibiotic concentration, and fit using the following equation:

$$\mu _m = \frac{{\mu IC_{50}^n}}{{A^n + IC_{50}^n}}$$

as previously described [48, 49]. All subsequent experiments that utilized carbenicillin concentrations were based on IC_50_ values from the control strain (Fig. 4A bottom, gray line) of 1.24 μg/mL.

### Pairwise competition experiments

The selected strains were grown overnight as previously described. In all cases, overnight cultures were resuspended in either LB or M9CAG media with 1 mM IPTG and appropriate antibiotic, and placed in 25 °C for one hour to initiate gene expression. Cells were then diluted 1:10,000, and 200 μL was added to wells in a microtiter plate surrounded by Milli-Q water. For competitions with carbenicillin, 0.93 μg/mL (75% IC_50_) was added directly to the well. The plate was then sealed with a paper film, covered with a plastic lid and incubated at 37 °C for 24 h with agitation at 250 rpm. Both at time = 0, and after 24 h, CFU was taken by serially diluting the cultures and plating 20 μL from appropriate dilutions onto agar plates in technical replicates of six. Plates were then grown for 16 h at 37 °C. Dilutions were chosen based on achieving between 10–70 colonies. Relative fitness (W) was quantified as described previously [50, 51], according to the following equation: W = log(M_0_/M_24_)/log(W_0_/W_24_), where 0 or 24 indicates the CFU at the corresponding time point, and M and W (“mutant” and “wild-type” respectively by convention) designate the two competing strains, as defined in Table S12. In all cases, the single-gene variant was designated as M. To differentiate strains from one another, we used two methods. First, we utilized identical DH5αPro hosts carrying plasmid variants containing either kanamycin resistance (kanR) or chloramphenicol resistance (cmR) genes. In these cases, 24 h competitions were conducted in the absence of any antibiotic selection, and M and W were each determined by CFU obtained from kanamycin or chloramphenicol-containing agar plates, respectively. We verified that plasmid loss was negligible over the 24-hour period in the absence of any selection for both strains (Fig. S5A*p* > 0.1, 2-tailed student *t* test), and that direct competition resulted in no statistical difference from 1 (Fig. S5B, *p* > 0.1, 2-tailed student *t* test). For the second method, all kanR plasmids were used, and instead changed the hosts such that DH5αPro cells were in competition with DH5αPro containing a spontaneous rifampicin-resistant mutant (rifR). Any rifR strain was quantified on rifampicin-containing plates, and the second strain was quantified by rifampicin CFU minus CFU obtained on blank plates. We established that rifR exhibited no fitness defects by (1) growth rates between the wild-type (WT) strain (W) and rifR (M) (Fig. S5D), and (2) directly competing the two control strains (Fig. S5E). In both cases, results were statistically indistinguishable (*p* > 0.1, two-tailed student *t* test). KanR/cmR and WT/rifR experiments were each conducted in LB or M9CAG, respectively. In all cases, experiments were repeated with at least three independent biological replicates.

### Time-kill measurements in the presence of carbenicillin

All strains were grown as previously described. Time-kill experiments entailed hourly measurements of CFU in presence of carbenicillin at either 3.75 μg/mL (3x IC_50_) or 5 μg/mL (4x IC_50_) over a span of 2 or 3 h, including time 0. Specifically, overnight cultures were first diluted 1:100 into LB media containing 1 mM IPTG and 50 μg/mL kanamycin and sub-cultured for two hours in a 37 °C incubator with shaking at 250 rpm. Following this, cell density was adjusted as necessary to achieve a starting OD_600_ of ~0.15 in all cases. Adjusted subcultures were then aliquoted into a 96-well plate and the appropriate carbenicillin treatments were added directly to the well. Plates were sealed with a paper film and placed in a 37 °C incubator with shaking at 250 rpm. Initial collection for time=0 was acquired before carbenicillin treatment. Thereafter, 10 μL of culture was removed from the well every hour, 10-fold serial dilutions were performed and 10 μL was plated on blank LB agar with three technical replicates at each time point. Colonies were counted after plates were grown for 16 h in a 37 °C incubator to determine CFU. This procedure utilized 14 strains of DH5αPro transformed with kanR plasmids of interest – ctrl, katG, lpxM, yfbR, aroH, pld, fdtC, agp, eptC, arcA, argF, mmuM, ahr, and fabG. CFUs were averaged for all technical replicates, and experiments were conducted with at least three independent biological replicates.

### Oxygen consumption rate

Oxygen consumption rates (OCR) were obtained with the Resipher device from Lucid Scientific. The selected strains were grown overnight as previously described. Overnight cultures were resuspended in M9CAG media with 1 mM IPTG and 50 μg/mL kanamycin, and placed in 25 °C for one hour to initiate gene expression. Following this, cells were diluted 10x into M9CAG media containing kanamycin and IPTG, and 100 μL was aliquoted per well into a 96-well microtiter plate according to the manufacturer’s instructions. Plates were placed at 30 °C to minimize growth, and oxygen concentration (μM) was measured immediately thereafter. 24 wells were measured consisting of 6 technical replicates for each strain. Given the clear well-well variability (Fig. S8B, C), data shown are for one biological replicate. However, qualitative trends were consistently reproduced in multiple independent experiments.

### Statistics

In all cases where *t* tests and ANOVA’s were used, data was first verified to be normally distributed using Kolmogorov test for normality. Otherwise, Mann-Whitney U-tests were conducted. For panels with multiple tests, Bonferroni correction was used to adjust the *p* values. To determine whether any metabolic category was significantly dependent on incompatibility groups, we implemented logistic regressions in MATLAB with the function fitglm. Random forest classification was used to establish the relative importance of prevalent metabolic genes and gene categories predicting the presence of antibiotic resistance genes. Chi-square tests were conducted to determine significant co-occurrence of individual antibiotic resistant and metabolism genes. Dissociative relationships were distinguished by the odds ratios from the chi-square tests. To investigate whether the strong associations and disassociations were driven by evolutionary constraints, or simply artifacts of a common ancestor, we re-ran our statistical analysis using Coinfinder [29] to take in our gene presence-absence data, along with the genome phylogeny, and compute the Bonferroni-corrected statistical likelihood of coincidence (either associations or dissociations), thereby accounting for evolutionary relatedness.

## Supplementary information

Supplementary Material

Table S1

Table S2

Table S3

Table S4

Table S5

Table S8

Table S9

Table S10

Table S11

Table S14

Table S13

## Data Availability

All sequencing data is publicly available according to the accession IDS listed in Supplementary Tables S2-3. All experimental raw data is available in the supplementary materials. Any other materials will be made available upon request.

## References

[1] Bottery MJ, Pitchford JW, Friman VP (2021). Ecology and evolution of antimicrobial resistance in bacterial communities. ISME J.

[2] Alonso-del Valle A, León-Sampedro R, Rodríguez-Beltrán J, DelaFuente J, Hernández-García M, Ruiz-Garbajosa P (2021). Variability of plasmid fitness effects contributes to plasmid persistence in bacterial communities. Nat Commun.

[3] Millan AS (2018). Evolution of plasmid-mediated antibiotic resistance in the clinical context. Trends Microbiol.

[4] Bennett PM (2008). Plasmid encoded antibiotic resistance: acquisition and transfer of antibiotic resistance genes in bacteria. Br J Pharm.

[5] Barlow M (2009). What antimicrobial resistance has taught us about horizontal gene transfer. Methods Mol Biol.

[6] Che Y, Xia Y, Li A-D, Yang Y, Zhang T (2019). Mobile antibiotic resistome in wastewater treatment plants revealed by nanopore metagenomic sequencing. Microbiome.

[7] van Hoek AHAM, Mevius D, Guerra B, Mullany P, Roberts PA, Aarts HJM (2011). Acquired antibiotic resistance genes: an overview. Front Microbiol.

[8] Shaikh S, Fatima J, Shakil S, Rizvi SM, Kamal MA (2015). Antibiotic resistance and extended spectrum beta-lactamases: types, epidemiology and treatment. Saudi J Biol Sci.

[9] Rodríguez-Martínez JM, Machuca J, Cano ME, Calvo J, Martínez-Martínez L, Pascual A (2016). Plasmid-mediated quinolone resistance: two decades on. Drug Resist Updat.

[10] Roer L, Overballe-Petersen S, Hansen F, Schønning K, Wang M, Røder BL (2018). *Escherichia coli* sequence type 410 is causing new international high-risk clones. mSphere.

[11] Zong Z, Fenn S, Connor C, Feng Y, McNally A (2018). Complete genomic characterization of two *Escherichia coli* lineages responsible for a cluster of carbapenem-resistant infections in a Chinese hospital. J Antimicrob Chemother.

[12] Dunn SJ, Connor C, McNally A (2019). The evolution and transmission of multi-drug resistant *Escherichia coli* and *Klebsiella pneumoniae*: the complexity of clones and plasmids. Curr Opin Microbiol.

[13] Carattoli A (2009). Resistance plasmid families in Enterobacteriaceae. Antimicrob Agents Chemother..

[14] Carattoli A (2013). Plasmids and the spread of resistance. Int J Med Microbiol.

[15] Rozwandowicz M, Brouwer MSM, Fischer J, Wagenaar JA, Gonzalez-Zorn B, Guerra B (2018). Plasmids carrying antimicrobial resistance genes in Enterobacteriaceae. J Antimicrob Chemother.

[16] Chen K, Xu X, Zhang L, Gou Z, Li S, Freilich S (2015). Comparison of four *Comamonas* catabolic plasmids reveals the evolution of pBHB to catabolize haloaromatics. Appl Environ Mircobiol.

[17] Schlüter A, Heuer H, Szczepanowski R, Forney LJ, Thomas CM, Pühler A (2003). The 64 508 bp IncP-1beta antibiotic multiresistance plasmid pB10 isolated from a waste-water treatment plant provides evidence for recombination between members of different branches of the IncP-1beta group. Microbiology.

[18] Yang JH, Wright SN, Hamblin M, McCloskey D, Alcantar MA, Schrübbers L (2019). A white-box machine learning approach for revealing antibiotic mechanisms of action. Cell.

[19] Lobritz MA, Belenky P, Porter CBM, Gutierrez A, Yang JH, Schwarz EG (2015). Antibiotic efficacy is linked to bacterial cellular respiration. Proc Natl Acad Sci USA.

[20] Lopatkin AJ, Bening SC, Manson AL, Stokes JM, Kohanski MA, Badran A (2021). Clinically relevant mutations in core metabolic genes confer antibiotic resistance. Science.

[21] Brynildsen MP, Winkler JA, Spina CS, MacDonald IC, Collins JJ (2013). Potentiating antibacterial activity by predictably enhancing endogenous microbial ROS production. Nat Biotechnol.

[22] Kohanski MA, Dwyer DJ, Hayete B, Lawrence CA, Collins JJ (2007). A Common mechanism of cellular death induced by bactericidal antibiotics. Cell.

[23] Guédon G, Libante V, Coluzzi C, Payot S, Leblond-Bourget N (2017). The obscure world of Integrative and mobilizable elements, highly widespread elements that pirate bacterial conjugative systems. Genes.

[24] Robertson J, Nash JHE (2018). MOB-suite: software tools for clustering, reconstruction and typing of plasmids from draft assemblies. Micro Genom.

[25] Weiss B (2007). The deoxycytidine pathway for thymidylate synthesis in *Escherichia coli.*. J Bacteriol.

[26] Clementz T, Zhou Z, Raetz CR (1997). Function of the *Escherichia coli MsbB* gene, a multicopy suppressor of *htrB* knockouts, in the acylation of lipid A. acylation by *MsbB* follows laurate incorporation by *HtrB*. J Biol Chem.

[27] Hillar A, Peters B, Pauls R, Loboda A, Zhang H, Mauk AG (2000). Modulation of the activities of catalase-peroxidase HPI of Escherichia coli by site-directed mutagenesis. Biochem.

[28] Kempes CP, van Bodegom PM, Wolpert D, Libby E, Amend J, Hoehler T (2017). Drivers of bacterial maintenance and minimal energy requirements. Front Microbiol.

[29] Whelan FJ, Rusilowicz M, McInerney JO (2020). Coinfinder: detecting significant associations and dissociations in pangenomes. Micro Genom.

[30] Dwyer DJ, Belenky PA, Yang JH, MacDonald CI, Martell JD, Takahashi N (2014). Antibiotics induce redox-related physiological alterations as part of their lethality. Proc Natl Acad Sci USA.

[31] Zhang Y, Heym B, Allen B, Young D, Cole S (1992). The catalase-peroxidase gene and isoniazid resistance of *Mycobacterium tuberculosis*. Nature.

[32] Musser JM (1995). Antimicrobial agent resistance in mycobacteria: molecular genetic insights. Clin Microbiol Rev.

[33] Lopatkin AJ, Stokes JM, Zheng EJ, Yang JH, Takahashi MK, You L (2019). Bacterial metabolic state more accurately predicts antibiotic lethality than growth rate. Nat Microbiol.

[34] Baquero F, Coque TM, de la Cruz F (2011). Ecology and evolution as targets: the need for novel eco-evo drugs and Strategies to fight antibiotic resistance. Antimicrob Agents Chemother.

[35] Antipov D, Hartwick N, Shen M, Raiko M, Lapidus A, Pevzner PA (2016). PlasmidSPAdes: assembling plasmids from whole genome sequencing data. Bioinformatics.

[36] Seemann T (2014). Prokka: rapid prokaryotic genome annotation. Bioinformatics.

[37] Kanehisa M, Goto S (2000). KEGG: kyoto encyclopedia of genes and genomes. Nucleic Acids Res.

[38] AMRFinderPlus - Pathogen Detection - NCBI. 2022. https://www.ncbi.nlm.nih.gov/pathogens/antimicrobial-resistance/AMRFinder/.

[39] Bortolaia V (2020). ResFinder 4.0 for predictions of phenotypes from genotypes. J Antimicrob Chemother.

[40] Carattoli A, Hasman H (2020). PlasmidFinder and in Silico pMLST: identification and typing of plasmid replicons in whole-genome requencing (WGS). Methods Mol Biol.

[41] Johansson MHK, Bortolaia V, Tansirichaiya S, Aarestrup FM, Roberts AP, Petersen TN (2021). Detection of mobile genetic elements associated with antibiotic resistance in *Salmonella enterica* using a newly developed web tool: MobileElementFinder. J Antimicrob Chemother.

[42] Page AJ, Cummins CA, Hunt M, Wong VK, Reuter S, Holden MTG (2015). Roary: rapid large-scale prokaryote pan genome analysis. Bioinformatics.

[43] Stamatakis A (2014). RAxML version 8: a tool for phylogenetic analysis and post-analysis of large phylogenies. Bioinformatics.

[44] Yu G (2020). Using Ggtree to visualize data on tree-like structures. Curr Protoc Bioinforma.

[45] Yu G, Lam TT-Y, Zhu H, Guan Y (2018). Two methods for mapping and visualizing associated data on phylogeny using Ggtree. Mol Bol Evol.

[46] Yu G, Smith DK, Zhu H, Guan Y, Lam TT-Y (2017). Ggtree: an R package for visualization and annotation of phylogenetic trees with their covariates and other associated data. Methods Ecol Evol.

[47] Zwietering MH, Jongenburger I, Rombouts FM, van’t Riet K (1990). Modeling of the bacterial growth curve. Appl Environ Microbiol.

[48] Lopatkin AJ, Huang S, Smith RP, Srimani JK, Sysoeva TA, Bewick S (2016). Antibiotics as a selective driver for conjugation dynamics. Nat Microbiol.

[49] Lopatkin AJ, Meredith HR, Srimani JK, Pfeiffer C, Durrett R, You L (2017). Persistence and reversal of plasmid-mediated antibiotic resistance. Nat Commun.

[50] Lin W, Zeng J, Wan K, Lv L, Guo L, Li X (2018). Reduction of the fitness cost of antibiotic resistance caused by chromosomal mutations under poor nutrient conditions. Environ Int..

[51] Lenski RE, Rose MR, Simpson SC, Tadler SC (1991). Long-term experimental evolution in *Escherichia coli. I*. adaptation and divergence during 2,000 generations. Am Nat.

