# Supplementary material for: Metabolic genes on conjugative plasmids are highly prevalent in Escherichia coli and can protect against antibiotic treatment
Source: ISME J. 2022 Oct 19;17(1):151–62. doi: 10.1038/s41396-022-01329-1 (PMC9750983; doi:10.1038/s41396-022-01329-1)
Supplement: Supplementary file 1 — Supplementary Material [file 41396_2022_1329_MOESM1_ESM.docx]

**Supplementary information for**

**Metabolic genes on conjugative plasmids are highly prevalent in *Escherichia coli* and can protect against antibiotic treatment**

Alana Palomino^1^, Danya Gewurz^1^, Lela DeVine^1^, Ujana Zajmi^1^, Jenifer Moralez^1^, Fatima Abu-Rumman^2^, Robert P. Smith^3^, and Allison J. Lopatkin^1,4-6^

^1^Department of Biology, Barnard College; New York, NY 10027; USA

^2^Department of Biological Sciences, Halmos College of Arts and Science, Nova Southeastern University; Fort Lauderdale, FL 33314; USA

^3^Cell Therapy Institute, Kiran Patel College of Allopathic Medicine, Nova Southeastern University; Fort Lauderdale, FL 33314; USA

^4^Department of Ecology, Evolution, and Environmental Biology, Columbia University; New York, NY 10027; USA

^5^Data Science Institute, Columbia University; New York, NY 10027; USA

^6^Department of Systems Biology, Columbia University; New York, NY 10027; USA

^*^Corresponding author: alopatkin@barnard.edu

**This PDF file includes:**

Figures S1 to S8

Tables S1 to S14

**Supplementary Figure 1: Inclusion criteria for *E. coli* isolates and sequence types (STs) used in dataset.**

Flow chart for systematic literature search. Articles were identified from PubMed and EMBASE and filtered by inclusion criteria to generate a high-quality dataset of expected *E. coli* isolates and STs.

**
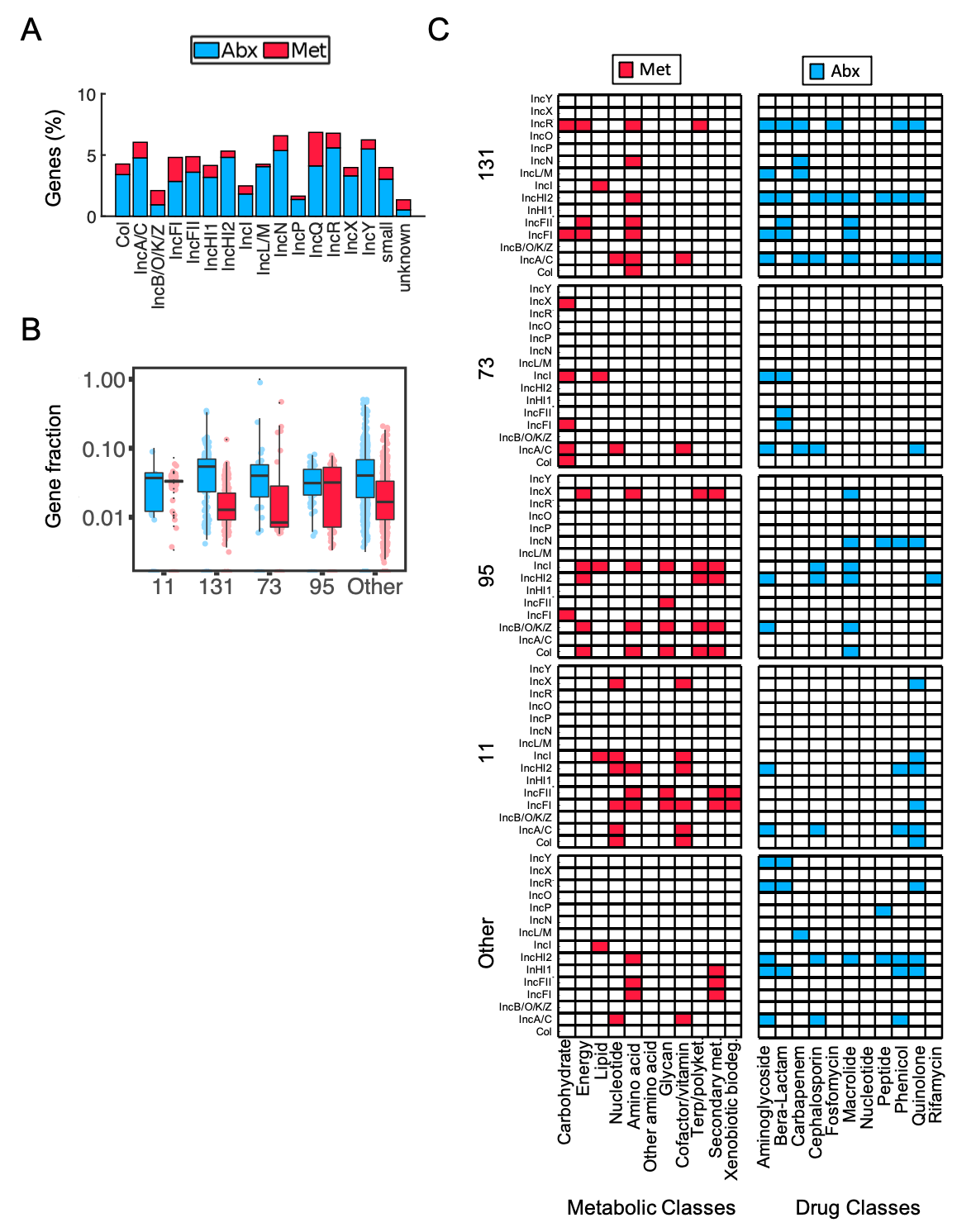
**

**Supplementary Figure 2: Incompatibility group breakdown and logistic regression.**

1. Gene breakdown per incompatibility group. Percentage of metabolic genes (red) and antibiotic resistance genes (blue) belonging to 15 known (17 total) incompatibility groups.
2. Gene proportion per ST. Proportion of metabolic genes (red) and antibiotic resistance genes (blue) belonging each prevalent (131, 11, 73, 95) or other ST.
3. Logistic regression heat map. Significant relationships between incompatibility groups and KEGG metabolic classes (red, *left*) or drug classes (blue, *right*), broken down by ST (131, 11, 73, 95 and other). Colored boxes indicate statistical significance (*p* < 0.05).

**
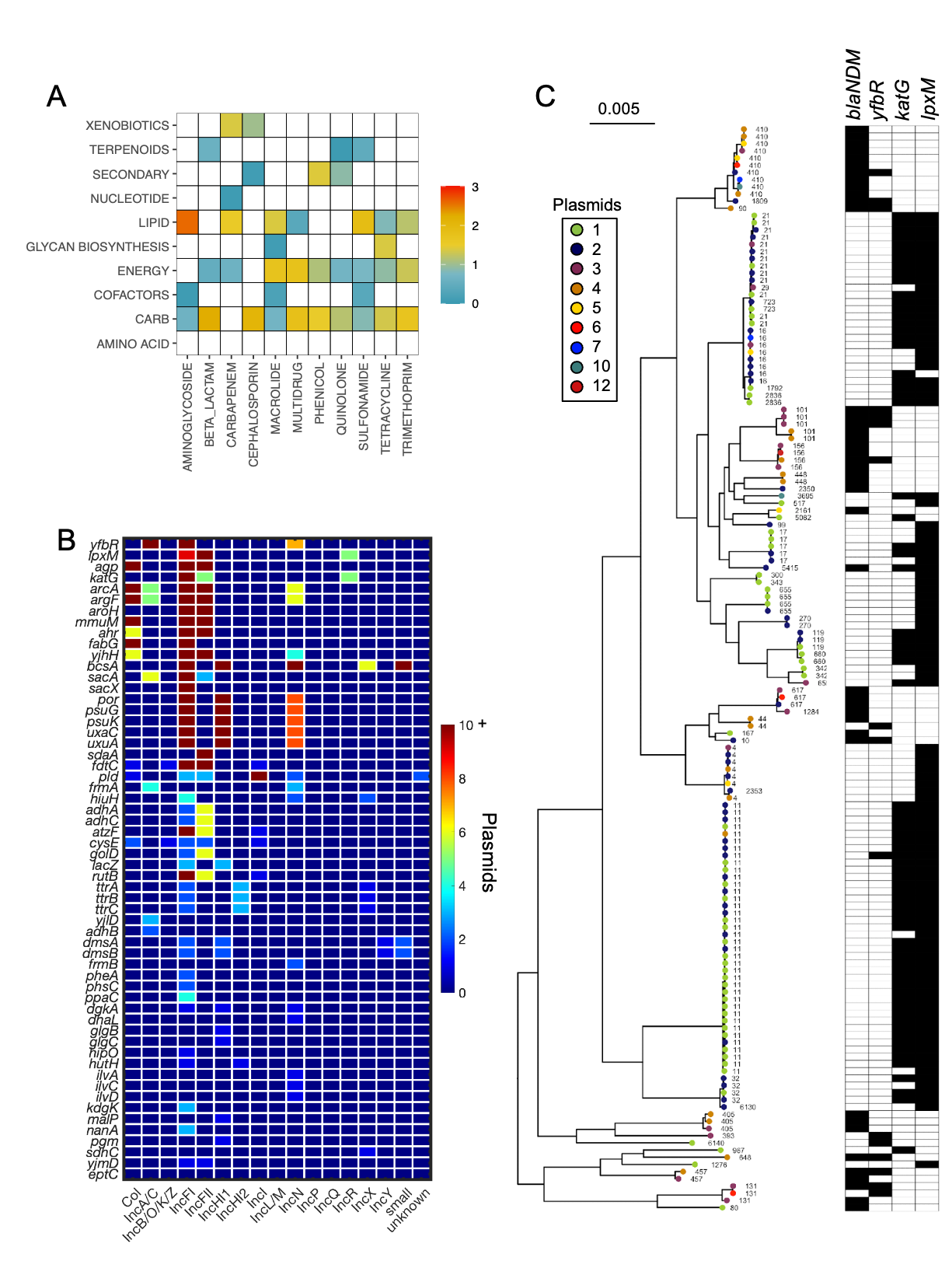
Supplementary Figure 3: Random forest out-of-bag predictor for metabolic class and antibiotic resistance class associations.**

1. Random forest classification. Heat map shows predicted correlations between known antibiotic classes and associated KEGG metabolic classes. Blue colors indicate little correlation, yellow colors indicate some correlation, and orange to red colors indicate high correlation between the two classes.
2. Phylogenetic analysis of STs carrying *blaNDM*, *katG*, *lpxM* and *yfbR* genes. This tree includes all strains carrying at least one of the four genes. Only strains classified as non-clinical were filtered out. The scale bar represents 0.005 nucleotide substitutions per site of the entire core genome. Tip colors represent the number of plasmids carried by each strain, and labeled by ST. Gene presence or absence is indicated by black or white, respectively.
3. Heat map of number of incompatibility groups where metabolic-antibiotic resistance gene associations occur. Every row represents a metabolic gene (e.g., Fig. 3A) with at least one associated antibiotic resistance gene. Columns represent each unique incompatibility group. The color intensity represents the number of plasmids corresponding to each metabolic association.

**Supplementary Figure 4: Minimum inhibitory concentrations (MICs) of carbenicillin for strains exogenously expressing metabolic genes of interest identified in our analysis**

1. MIC for IPTG-induced blaNDM (dotted line) compared to IPTG-free control at 11 concentrations of carbenicillin. Error bars indicate at least three biological replicates.
2. MIC for ctrl, katG, lpxM, and yfbR (gray, green, purple, and orange, respectively) with carbenicillin. Error bars indicate at least three replicates of each.

**
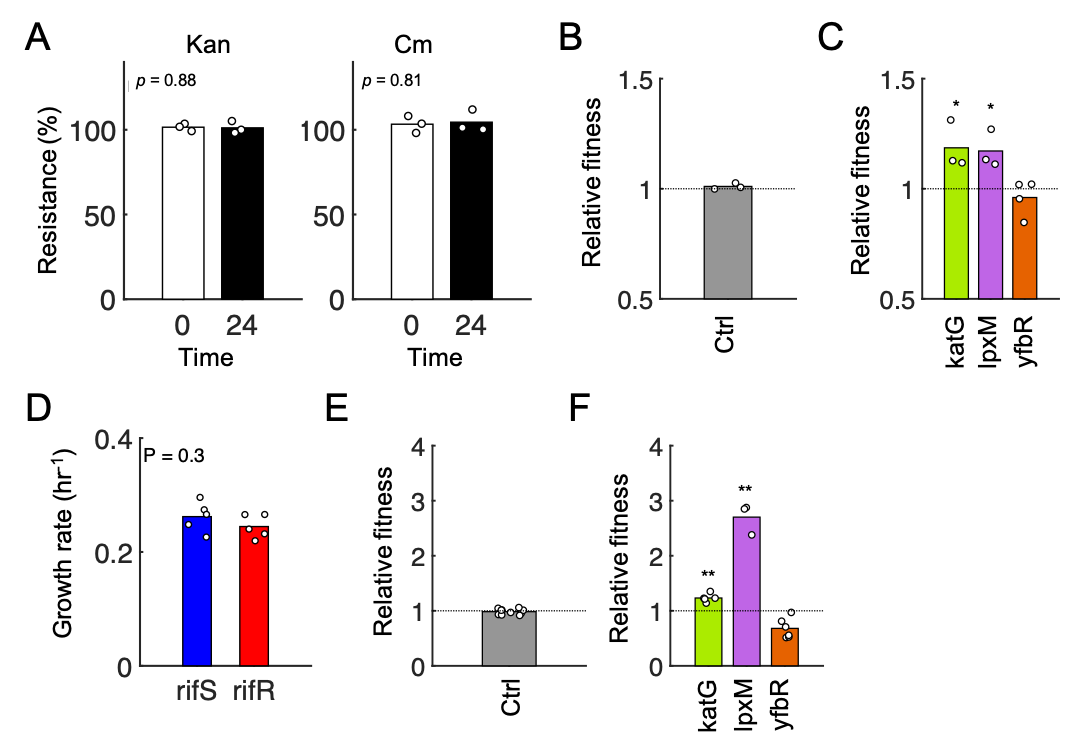
Supplementary Figure 5: Relative fitness advantage of dissociated metabolic genes**

1. Plasmid loss control for ctrl-kanR strain (*left*, pLac/Ara-kanR, S2 in Table S6B) and ctrl-cmR strain (*right*, pLac/Ara-cmR, S19 in Table S6B). Overnight cultures of each strain were grown in the presence of the respective drug. Then, each strain was grown individually under conditions identical to competition experiments (B) in LB media (i.e., no antibiotic). Percentage of the population carrying the plasmid was calculated by comparing CFU on antibiotic-containing agar plates to blank agar plates at time 0 and time 24. *p-values* are obtained from two-tailed student t-tests; bars are averages from three independent biological replicates.
2. ctrl-kanR competion against ctrl-cmR in LB media. Relative fitness is calculated as ctrl-kanR to ctrl-cmR, and is statistically identical to 1 (*p* > 0.5).
3. Relative fitness of metabolic-antibiotic resistance gene (dis)associations. katG and lpxM are both more fit compared to katG-blaNDM and lpxM-blaNDM, respectively (one star, *p* < 0.05). yfbR is statistically identical to yfbR-blaNDM (*p* > 0.1). Single genes are on kanamycin resistant plasmids and dual genes on chloramphenicol resistant plasmids.
4. Growth comparison between rifR and rifS strains (S1and S10 in Table S6B). Growth rates are statistically insignificantly different (*p* > 0.1).
5. Competition between rifR-ctrl and rifS-ctrl strains (S2 and S11 in Table S6B); relative fitness is not statistically different from 1 (*p* > 0.1).
6. Relative fitness of metabolic-antibiotic resistance gene (dis)associations. katG and lpxM are both more fit compared to katG-blaNDM and lpxM-blaNDM, respectively (two stars, *p* < 0.01). yfbR is statistically identical to yfbR-blaNDM (*p* > 0.1). All genes are on kanamycin resistant plasmids; single genes are in rifS hosts and dual genes in rifR hosts.

**
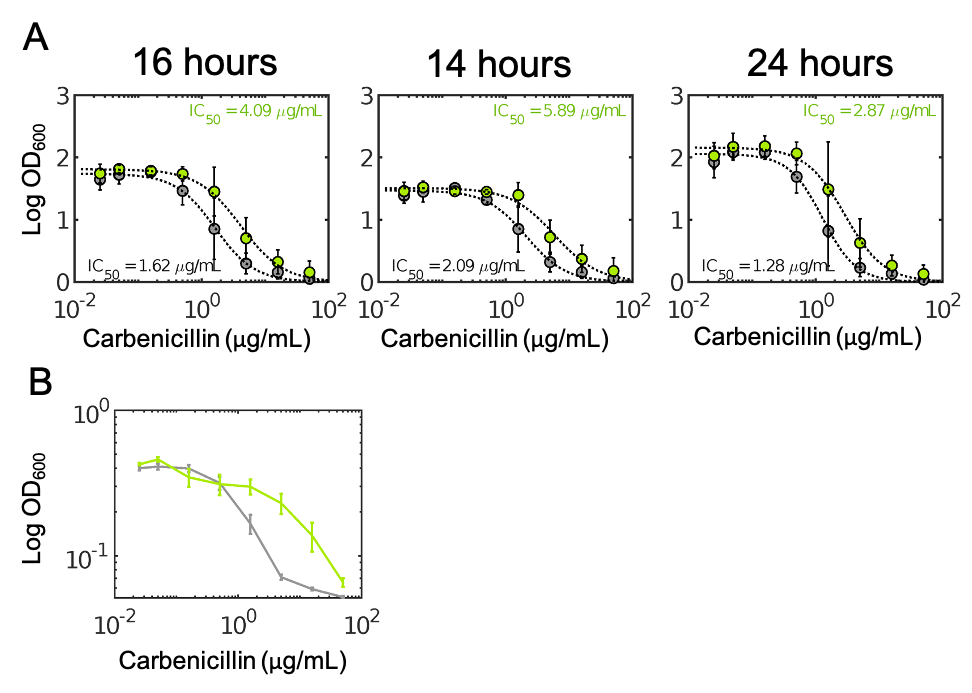
Supplementary Figure 6: Comparing temporal dynamics between katG and control strains.**

1. IC_50_ values determined from cell density (OD_600_) at 14, 16, and 24 hours. Green indicates katG and gray indicates ctrl. X-axis is carbenicillin concentration in μg/mL and y-axis is log OD_600_. Error bars represent at least three independent replicates.
2. Log OD_600_ values of katG (green) and ctrl (grey) after 20 hours of growth in the absence of mineral oil. X-axis is carbenicillin concentration in μg/mL and y-axis is log OD_600_. Error bars represent at least three independent replicates.


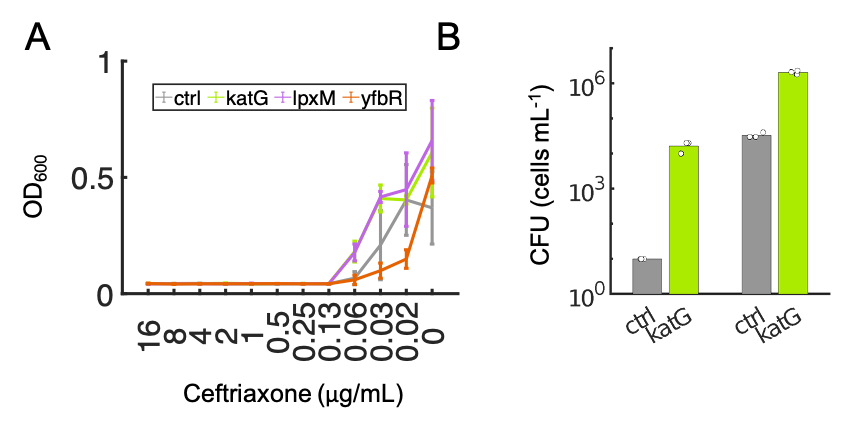


**Supplementary Figure 7: Insights into additional MIC and colony forming unit (CFU) trends.**

1. MICs for metabolic and control strains with ceftriaxone. OD_600_ values for katG, lpxM, and yfbR were compared with ctrl at 11 different concentrations of ceftriaxone and one antibiotic free control. Error bars indicated standard deviation.
2. CFUs for ctrl and katG. The condition corresponding to the ceftriaxone MIC of ctrl in (A) (~0.06 μg/mL) was used to measure CFU for two biological replicates of katG and ctrl.

**
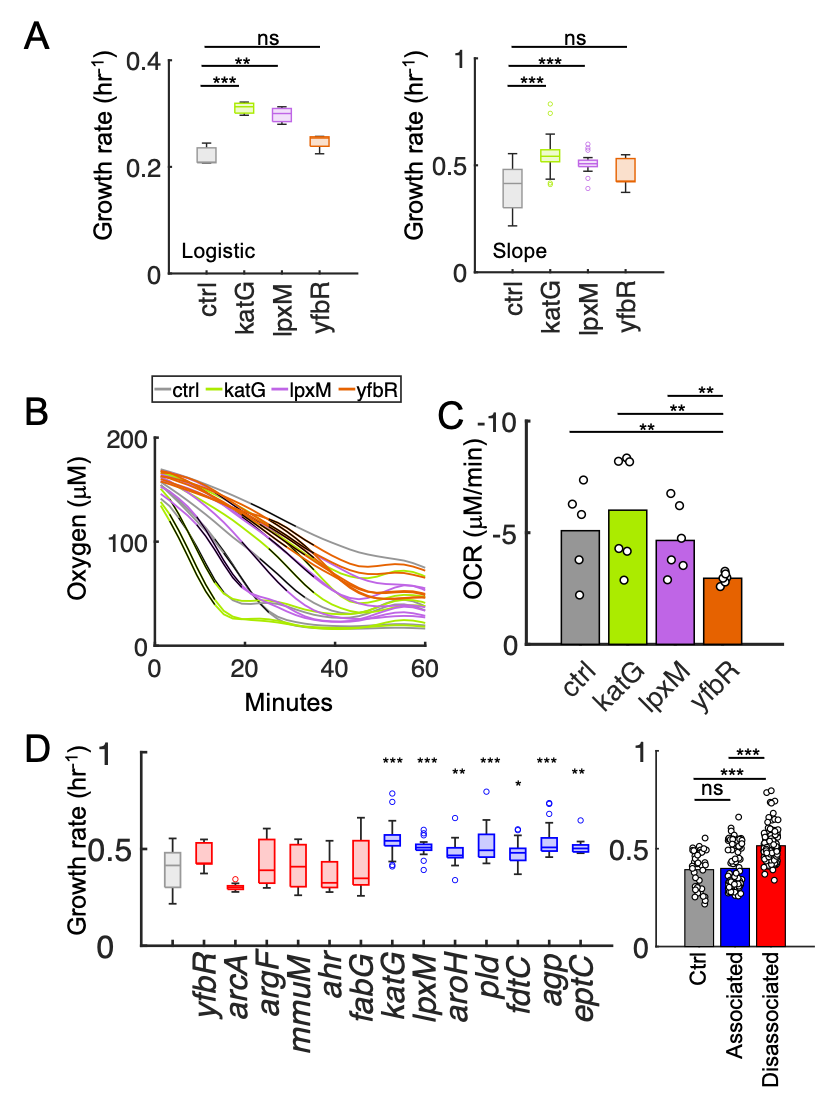
Supplementary Figure 8: Oxygen consumption rates (OCR) and growth characterization.**

1. Growth rates in rich media (LB). Boxes represent averages of at least 3 independent biological replicates. Left and right panels were quantified using the logistic or maximum derivative (i.e., slope) methods, respectively, as described in the Methods section.
2. Oxygen concentration over time. O_2_ levels obtained from the Resipher probe were captured in intervals of 30 seconds for one hour. Black lines indicate temporal intervals over which rates were determined.
3. OCR calculated from (B). yfbR is significantly less than three other strains (*p* < 0.005, one-tailed t-test).
4. Growth rates in rich (LB) media. *Left*: Growth rates for all 5 associated (red) genes, 6 disassociated genes (blue), and ctrl (gray). Boxes represent averages of at least 3 independent biological replicates. All disassociated (blue) genes are statistically different from ctrl using multiple comparison of the means with Bonferroni correction (*p* < 0.05); in contrast, all associated (red) genes are statistically indistinguishable from ctrl. *Right*: Pooled data from the left panel remains consistent. See Table S13 for statistics.

One, two and three asterisks indicate *p* < 0.05, 0.01, and 0.001, respectively, and ns is not significant, where applicable. All *p-values* from panels with multiple t-tests were Bonferroni-corrected

**Table S1 (separate file): Literature search ST data used in this study.**

Unique STs, the number of times each ST occurred, as well as the corresponding percentage, as determined from a systematic literature search.

**Table S2 (separate file): Plasmid overview dataset used in this study.**

Complete list of downloaded plasmids and associated metadata before filtering.

**Table S3 (separate file): Comprehensive plasmid genome dataset used in this study.**

Final set of conjugative and mobilizable plasmids, along with relevant metadata and annotations, used for analysis in this study.

**Table S4 (separate file): Gene association and dissociation statistics.** A-E are separated into individual sheets.

1. Each metabolic and statistically related antibiotic resistance gene, along with drug class it confers resistance to, are labeled as associated or disassociated. *p-values* and odds ratios are included for statistical backing behind designation.
2. Gene names listed in descending order by cumulative number of significant (dis)associations with antibiotic resistance genes. Separate columns contain the number of significant (dis)associations per gene.
3. Full statistical summary of all gene pairs.
4. Number of incompatibility groups associated with every gene pair.
5. Coinfinder statistics for gene associations and disassociations based on Fig. S3C phylogeny. Rows in yellow highlight significant pairs.

**Table S5 (separate file): Gene association and dissociation for plasmid-encoded mobile elements**

1. Mobile elements associated with *yfbR*, *blaNDM*, *lpxM*, and *katG*.
2. Number of each gene associated with a mobile element compared to the total number in the dataset. Genes are concatenated with semicolons if both were found on the same mobile element; in these cases, gene start locations are also concatenated in the same order as the listed genes and refers to the start position of the gene on the corresponding plasmid.

**Table S6: Strains and primers used to create transgenic strains in this study.**

1. Primer names and sequences are listed in table format. This includes pertinent information on which primers were used for the purposes of generating transgenic strains and what their sequences are.

| Primer name | Sequence (5’ -> 3’) |
| --- | --- |
| YfbR forward | TTAAGGTACCCATGAAACAGAGCCATTTTTTTGCCCATCTCTC |
| YfbR reverse | GGCCAAGCTTTTACAGCGGTGAATCCTGGCTAATCTCATCG |
| IpxM forward | TTAAGGTACCCATGGAAACGAAAAAAAATAATAGCGAATACATTCCTGAGT |
| IpxM reverse | GGCCAAGCTTTTATTTGATGGGATAAAGATCTTTGCGCTTATACGGC |
| KatG forward | TTAAGGTACCCATGAGCACGTCAGACGATATCCATAACACCAC |
| KatG reverse | GGCCAAGCTTTTACAGCAGGTCGAAACGGTCGAG |
| AroH forward | TTAAGAATTCATTAAAGAGGAGAAAGGTACCCATGAACAGAACTGACGAACTCCGTACT |
| AroH reverse | TTAAAAGCTTTCAGAAGCGGGTATCTACCGCA |
| Agp forward | TTAAGGTACCCATGAACAAAACGCTAATCGCCGC |
| Agp reverse | TTAATCTAGATTATTTCACCGCTTCATTCAACACGCTATC |
| ArcA forward | TTAAGGTACCCATGCAGACCCCGCACATTCTTATC |
| ArcA reverse | TTAAAAGCTTTTAATCTTCCAGATCACCGCAGAAGC |
| ArgF forward | TTAAGGTACCCATGTCCGATTTATACAAAAAACACTTTCTGAAACTG |
| ArgF reverse | TTAATCTAGATCACTCCCCAAGCGTTGC |
| MumM forward | TTAAGGTACCCATGTCGCAGAATAATCCGTTACGCG |
| MumM reverse | TTAAAAGCTTTCAGCTTCGCGCTTTTAACGC |
| Ahr forward | TTAAGGTACCCATGTCGATGATAAAAAGCTATGCCGC |
| Ahr reverse | TTAAAAGCTTTCAAAAATCGGCTTTCAACACCACG |
| FabG forward | TTAAGAATTCATTAAAGAGGAGAAAGGTACCCATGAATTTTG AAGGAAAAAT CGCACTG |
| FabG reverse | TTAAAAGCTTTCAGACCATGTACATCCCGC |
| FdtC forward | TTAAGGTACCCATGAAATTTATGGAATGTGC |
| FdtC reverse | TTAAAAGCTTTCATAATATTCTGAGCAGTCTTG |
| Pld forward | TTAAGGTACCCATGTCAGGTAAAAAAACTATCG |
| Pld reverse | TTAAAAGCTTTCAGGGTTTCCAGTCTGTAC |
| EptC forward | TTAA GGTACCC ATGCATTCCACAGAAGTCC |
| EptC reverse | TTAA AAGCTT TTACTGATTACCCACCTGATC |
| NDM-1 forward | TTAAGAATTCATTAAAGAGGAGAAAGGTACCCATGGAACTGCCGAACATT ATGCACCC |
| NDM-1 reverse | GGCCAAGCTTTCAACGCAGTTTGTCAGCCATACGG |

| **NDM-1 - metabolic gene fusions** | |
| --- | --- |
| Ybr HindIII Forward | TTAAAAGCTTATTAAAGAGGAGAAAGGTACCCATGAAACAGAGCCATTTTTT  TGCCCATCTCTC |
| Ybr reverse | GGCCAAGCTTTTACAGCGGTGAATCCTGGCTAATCTCATCG |
| IpxM HindIII Forward | TTAAAAGCTTATTAAAGAGGAGAAAGGTACCCATGGAAACGAAAAAAAATA ATAGCGAATACATTCCTGAGT |
| IpxM reverse | GGCCAAGCTTTTATTTGATGGGATAAAGATCTTTGCGCTTATACGGC |
| KatG HindIII Forward | TTAAAAGCTTATTAAAGAGGAGAAAGGTACCCATGAGCACGTCAGACGATA  TCCATAACACCAC |
| KatG reverse | GGCCAAGCTTTTACAGCAGGTCGAAACGGTCGAG |

1. Information for all strains and plasmids used in this study.

| **Number** | **Name** | **Strain** | **Plasmid** | **Description** | **Selection drug** |
| --- | --- | --- | --- | --- | --- |
| S1 | WT | DH5alphaPro |  | F^–^ *endA1* *glnV44* *thi-1* *recA1* *relA1* *gyrA96* *deoR* *nupG* *purB20* φ80d*lacZ*ΔM15 Δ(*lacZYA-argF*)U169, hsdR17(*r_K_*^–^*m_K_*^+^), λ^–^ |  |
| S2 | ctrl | DH5alphaPro | pLac/Ara | DH5αPRO carrying no gene under an IPTG-inducible promoter on a Kan^R^ plasmid | Kan |
| S3 | blaNDM | DH5alphaPro | pLac/Ara-blaNDM-1 | DH5αPRO carrying *blaNDM-1* under an IPTG-inducible promoter on a Kan^R^ plasmid | Kan |
| S4 | katG | DH5alphaPro | pLac/Ara-katG | DH5αPRO carrying *katG* under an IPTG-inducible promoter on a Kan^R^ plasmid | Kan |
| S5 | lpxM | DH5alphaPro | pLac/Ara-lpxM | DH5αPRO carrying *lpxM* under an IPTG-inducible promoter on a Kan^R^ plasmid | Kan |
| S6 | yfbR | DH5alphaPro | pLac/Ara-yfbR | DH5αPRO carrying *yfbR* under an IPTG-inducible promoter on a Kan^R^ plasmid | Kan |
| S7 | katG-blaNDM | DH5alphaPro | pLac/Ara-katG-blaNDM-1 | DH5αPRO carrying *katG-blaNDM-1* under an IPTG-inducible promoter on a Kan^R^ plasmid | Kan |
| S8 | lpxM-blaNDM | DH5alphaPro | pLac/Ara-lpxM-blaNDM-1 | DH5αPRO carrying *lpxM-blaNDM-1* under an IPTG-inducible promoter on a Kan^R^ plasmid | Kan |
| S9 | yfbR-blaNDM | DH5alphaPro | pLac/Ara-yfbR-blaNDM-1 | DH5αPRO carrying *yfbR-blaNDM-1* under an IPTG-inducible promoter on a Kan^R^ plasmid | Kan |
| S10 | rifR | DH5alphaPro-RifR |  | F^–^ *endA1* *glnV44* *thi-1* *recA1* *relA1* *gyrA96* *deoR* *nupG* *purB20* φ80d*lacZ*ΔM15 Δ(*lacZYA-argF*)U169, hsdR17(*r_K_*^–^*m_K_*^+^), λ^–^, Rif^R^ mutant | Rif |
| S11 | ctrl-rifR | DH5alphaPro-RifR | pLac/Ara | DH5αPRO rifR mutant carrying no gene under an IPTG-inducible promoter on a Kan^R^ plasmid | Rif, Kan |
| S12 | blaNDM-rifR | DH5alphaPro-RifR | pLac/Ara-blaNDM1 | DH5αPRO-rifR mutant carrying *blaNDM-1* under an IPTG-inducible promoter on a Kan^R^ plasmid | Rif, Kan |
| S13 | katG-rifR | DH5alphaPro-RifR | pLac/Ara-katG | DH5αPRO-rifR mutant carrying *katG* under an IPTG-inducible promoter on a Kan^R^ plasmid | Rif, Kan |
| S14 | lpxM-rifR | DH5alphaPro-RifR | pLac/Ara-lpxM | DH5αPRO-rifR mutant carrying *lpxM* under an IPTG-inducible promoter on a Kan^R^ plasmid | Rif, Kan |
| S15 | yfbR-rifR | DH5alphaPro-RifR | pLac/Ara-yfbR | DH5αPRO-rifR mutant carrying *yfbR* under an IPTG-inducible promoter on a Kan^R^ plasmid | Rif, Kan |
| S16 | katG-blaNDM-rifR | DH5alphaPro-RifR | pLac/Ara-katG-blaNDM-1 | DH5αPRO-rifR mutant carrying *katG-blaNDM-1* under an IPTG-inducible promoter on a Kan^R^ plasmid | Rif, Kan |
| S17 | lpxM-blaNDM-rifR | DH5alphaPro-RifR | pLac/Ara-lpxM-blaNDM-1 | DH5αPRO-rifR mutant carrying *lpxM-blaNDM-1* under an IPTG-inducible promoter on a Kan^R^ plasmid | Rif, Kan |
| S18 | yfbR-blaNDM-rifR | DH5alphaPro-RifR | pLac/Ara-yfbR-blaNDM-1 | DH5αPRO-rifR mutant carrying *yfbR-blaNDM-1* under an IPTG-inducible promoter on a Kan^R^ plasmid | Rif, Kan |
| S19 | ctrl-cmR | DH5alphaPro | pLac/Ara | DH5αPRO carrying no gene under an IPTG-inducible promoter on a Cm^R^ plasmid | Cm |
| S20 | blaNDM-1 | DH5alphaPro | pLac/Ara-blaNDM-1 | DH5αPRO carrying *blaNDM-1* under an IPTG-inducible promoter on a Cm^R^ plasmid | Cm |
| S21 | katG-blaNDM | DH5alphaPro | pLac/Ara-katG-blaNDM-1 | DH5αPRO carrying *katG* under an IPTG-inducible promoter on a Cm^R^ plasmid | Cm |
| S22 | lpxM-blaNDM | DH5alphaPro | pLac/Ara-lpxM-blaNDM-1 | DH5αPRO carrying *lpxM* under an IPTG-inducible promoter on a Cm^R^ plasmid | Cm |
| S23 | yfbR-blaNDM | DH5alphaPro | pLac/Ara-yfbR-blaNDM-1 | DH5αPRO carrying *yfbR* under an IPTG-inducible promoter on a Cm^R^ plasmid | Cm |
| S24 | aroH | DH5alphaPro | pLac/Ara-aroH | DH5αPRO carrying *aroH* under an IPTG-inducible promoter on a Cm^R^ plasmid | Kan |
| S25 | pld | DH5alphaPro | pLac/Ara-pld | DH5αPRO carrying *pld* under an IPTG-inducible promoter on a Cm^R^ plasmid | Kan |
| S26 | fdtC | DH5alphaPro | pLac/Ara-fdtC | DH5αPRO carrying *fdtC* under an IPTG-inducible promoter on a Cm^R^ plasmid | Kan |
| S27 | agp | DH5alphaPro | pLac/Ara-agp | DH5αPRO carrying *agp* under an IPTG-inducible promoter on a Cm^R^ plasmid | Kan |
| S28 | eptC | DH5alphaPro | pLac/Ara-eptC | DH5αPRO carrying *eptC* under an IPTG-inducible promoter on a Cm^R^ plasmid | Kan |
| S29 | arcA | DH5alphaPro | pLac/Ara-arcA | DH5αPRO carrying *arcA* under an IPTG-inducible promoter on a Cm^R^ plasmid | Kan |
| S30 | argF | DH5alphaPro | pLac/Ara-argF | DH5αPRO carrying *argF* under an IPTG-inducible promoter on a Cm^R^ plasmid | Kan |
| S31 | mmuM | DH5alphaPro | pLac/Ara-mmuM | DH5αPRO carrying *mmuM* under an IPTG-inducible promoter on a Cm^R^ plasmid | Kan |
| S32 | ahr | DH5alphaPro | pLac/Ara-ahr | DH5αPRO carrying *ahr* under an IPTG-inducible promoter on a Cm^R^ plasmid | Kan |
| S33 | fabG | DH5alphaPro | pLac/Ara-fabG | DH5αPRO carrying *fabG* under an IPTG-inducible promoter on a Cm^R^ plasmid | Kan |

Kan^R^: kanamycin resistance

Cm^R^: chloramphenicol resistance

1. Description of gene functions

|  | **Gene** | **Description** | **Direction** |
| --- | --- | --- | --- |
| 1 | *blaNDM* | Canonical β-lactamase | NA |
| 2 | *katG* | Catalase peroxidase | Dissociated |
| 3 | *lpxM* | Cell wall synthesis/ sigmaE regulated | Dissociated |
| 4 | *yfbR* | Nucleotide salvage/ sigma32 regulated | Associated |
| 5 | *aroH* | Amino acid synthesis enhanced under O_2_ limitation /sigma70 regulated | Dissociated |
| 6 | *pld* | Outer member/wall assembly/sigma70 regulated | Dissociated |
| 7 | *fdtC* | Glycan biosynthesis | Dissociated |
| 8 | *agp* | Growth on glucose-1-phosphatase | Dissociated |
| 9 | *eptC* | Outer member/wall assembly | Dissociated |
| 10 | *arcA* | Repression of genes involved in aerobic resp. | Associated |
| 11 | *argF* | Arginine biosynthesis/positively regulates catabalism with argR | Associated |
| 12 | *fabG* | Fatty acid biosynthesis/assoc. with plsX /anaerobic repr. | Associated |
| 13 | *mmuM* | Homocysteine S-methyltransferase | Associated |
| 14 | *ahr* | NADPH-dependent aldehyde reductase | Associated |

**Table S7: Growth inhibition antibiotic concentrations**

Concentrations of eight antibiotics in μg/mL. Seven logarithmically-spaced dilutions plus one antibiotic free control were used for growth inhibition experiments.

| Carbenicillin (μg/mL) | Ceftriaxone (μg/mL) | Chloramphenicol (μg/mL) | Gentamicin (μg/mL) | Streptomycin (μg/mL) | Ertapenem (μg/mL) | Ciprofloxacin (μg/mL) | Amoxicillin/clavulanate (μg/mL) |
| --- | --- | --- | --- | --- | --- | --- | --- |
| 50 | 16 | 50 | 50 | 50 | 10 | 1 | 10 |
| 15.8114 | 5.0596 | 15.8114 | 15.8114 | 15.8114 | 3.1623 | 0.3162 | 3.1623 |
| 5 | 1.6 | 5 | 5 | 5 | 1 | 0.1 | 1 |
| 1.5811 | 0.506 | 1.5811 | 1.5811 | 1.5811 | 0.3162 | 0.0316 | 0.3162 |
| 0.5 | 0.16 | 0.5 | 0.5 | 0.5 | 0.1 | 0.01 | 0.1 |
| 0.1581 | 0.0506 | 0.1581 | 0.1581 | 0.1581 | 0.0316 | 0.0032 | 0.0316 |
| 0.05 | 0.016 | 0.05 | 0.05 | 0.05 | 0.01 | 0.001 | 0.01 |
| 0 | 0 | 0 | 0 | 0 | 0 | 0 | 0 |

**Table S8 (separate file): Carbenicillin-mediated growth inhibition of katG relative to the control strain**

Raw data for katG and ctrl replicates under eight dilutions of carbenicillin treatment.

**Table S9 (separate file): Representative time-course data**

Cell densities for all three metabolic strains (katG, lpxM, and yfbR) and ctrl at 1.58 μg/mL carbenicillin over 24 hours.

**Table S10 (separate file): Density-based dose response data compiled**

Density after 20 hours for ctrl, katG, lpxM, and yfbR at least six biological replicates per strain for each antibiotic treatment. OD_600_ values shown for carbenicillin, ceftriaxone, amoxicillin/clavulanate, chloramphenicol, ciprofloxacin, ertapenem, gentamicin, and streptothricin.

**Table S11 (separate file): Colony forming unit (CFU) data on ctrl and katG strains**

CFUs for ctrl and katG strains that confirm MIC data.

**Table S12: Competition experiments**

Strain names, media, antibiotic, replicate number, and associated figure for all competition experiments.

| **#** | **M (strain 1)** | **W (strain 2)** | **Media** | **Carb** | **Replicates** | **Figure** |
| --- | --- | --- | --- | --- | --- | --- |
| 1 | ctrl (S2) (also called ctrl-kanR when compared with ctrl-cmR) | ctrl-rifR (S11) | M9CAG | 0 μg/mL | 15 clones | S5E |
| 2 | katG (S4) | katGblaNDM-rifR (S16) | M9CAG | 0 μg/mL | 3 clones | S5F |
| 3 | lpxM (S5) | lpxMblaNDM-rifR (S17) | M9CAG | 0 μg/mL | 3 clones | S5F |
| 5 | yfbR (S6) | yfbRblaNDM-rifR (S18) | M9CAG | 0 μg/mL | 3 clones | S5F |
| 6 | ctrl (S2) | ctrl-cmR (S11) | LB | 0 μg/mL | 3 clones | 5A left, S5B |
| 7 | katG (S4) | katGblaNDM-cmR (S21) | LB | 0 μg/mL | 3 clones | S5C |
| 8 | lpxM (S5) | lpxMblaNDM-cmR (S22) | LB | 0 μg/mL | 3 clones | S5C |
| 9 | yfbR (S6) | yfbRblaNDM-cmR (S23) | LB | 0 μg/mL | 3 clones | S5C |
| 10 | katG (S4) | ctrl-cmR (S11) | LB | 0 μg/mL | 3 clones | 5A left |
| 11 | katG (S4) | ctrl-cmR (S11) | LB | 0.93 μg/mL | 3 clones | 5A right |
| 12 | lpxM (S5) | ctrl-cmR (S11) | LB | 0 μg/mL | 3 clones | 5A left |
| 13 | lpxM (S5) | ctrl-cmR (S11) | LB | 0.93 μg/mL | 3 clones | 5A right |
| 14 | yfbR (S6) | ctrl-cmR (S11) | LB | 0 μg/mL | 3 clones | 5A left |
| 15 | yfbR (S6) | ctrl-cmR (S11) | LB | 0.93 μg/mL | 3 clones | 5A right |

**Table S13: One-way ANOVA results for growth rate comparisons**

1. Growth rate means and standard errors for each gene in rich media
2. One-way ANOVA pair-wise multiple comparison test results and *p-values* for growth rates

**Table S14: One-way ANOVA results for time-kill comparisons**

1. Survival means and standard errors for each gene at t = 2 hours and 3x IC_50_
2. One-way ANOVA pair-wise multiple comparison test results and *p-values* for at t = 2 hours and 3x IC_50_
3. Survival means and standard errors for each gene at t = 1 hour and 3x IC_50_
4. One-way ANOVA pair-wise multiple comparison test results and *p-values* for at t = 1 hour and 3x IC_50_
5. Survival means and standard errors for each gene at t = 1 hour and 4x IC_50_
6. One-way ANOVA pair-wise multiple comparison test results and *p-values* for at t = 1 hour and 4x IC_50_
